# Development and evaluation of deuterated [^18^F]JHU94620 isotopologues for the non-invasive assessment of the cannabinoid type 2 receptor in brain

**DOI:** 10.1186/s41181-024-00319-2

**Published:** 2024-12-23

**Authors:** Daniel Gündel, Mudasir Maqbool, Rodrigo Teodoro, Friedrich-Alexander Ludwig, Anne Heerklotz, Magali Toussaint, Winnie Deuther-Conrad, Guy Bormans, Peter Brust, Klaus Kopka, Rareş-Petru Moldovan

**Affiliations:** 1 Department of Experimental Neurooncological Radiopharmacy, Institute of Radiopharmaceutical Cancer Research, Helmholtz-Zentrum Dresden-Rossendorf, Research Site Leipzig, Permoserstrasse 15, 04318 Leipzig, Germany; 2 Molecular Imaging Branch, National Institute of Mental Health, National Institutes of Health, Bethesda, MD 20892-1026 USA; 3 Life Molecular Imaging GmbH, 13353 Berlin, Germany; 4 Radiopharmaceutical Research, Department of Pharmaceutical and Pharmacological Sciences, KU Leuven, BE-3000 Leuven, Belgium; 5 The Lübeck Institute of Experimental Dermatology, University Medical Center Schleswig-Holstein, 23562 Lübeck, Germany; 6 Faculty of Chemistry and Food Chemistry, School of Science, TU Dresden, 01069 Dresden, Germany

**Keywords:** Cannabinoid type 2 receptor, [^18^F]JHU94620, Neuroinflammation, Positron emission tomography

## Abstract

**Background:**

The cannabinoid type 2 receptors (CB2R) represent a target of increasing importance in neuroimaging due to its upregulation under various neuropathological conditions. Previous evaluation of [^18^F]JHU94620 for the non-invasive assessment of the CB2R availability by positron emission tomography (PET) revealed favourable binding properties and brain uptake, however rapid metabolism, and generation of brain-penetrating radiometabolites have been its main limitations. To reduce the bias of CB2R quantification by blood–brain barrier (BBB)-penetrating radiometabolites, we aimed to improve the metabolic stability by developing -*d*_4_ and -*d*_8_ deuterated isotopologues of [^18^F]JHU94620.

**Results:**

The deuterated [^18^F]JHU94620 isotopologues showed improved metabolic stability avoiding the accumulation of BBB-penetrating radiometabolites in the brain over time. CB2R-specific binding with *K*_D_ values in the low nanomolar range was determined across species. Dynamic PET studies revealed a CB2R-specific and reversible uptake of [^18^F]JHU94620-*d*_8_ in the spleen and to a local *h*CB2R(D80N) protein overexpression in the striatal region in rats.

**Conclusion:**

These results support further investigations of [^18^F]JHU94620-*d*_8_ in pathological models and tissues with a CB2R overexpression as a prerequisite for clinical translation.

**Supplementary Information:**

The online version contains supplementary material available at 10.1186/s41181-024-00319-2.

## Background

The cannabis plant *Cannabis sativa* is known for its psychoactive effects and used for various applications since ancient times (Fraguas-Sánchez and Torres-Suárez 2018; Alger 2013). The identification of the phytocannabinoids Δ^9^-*trans*-tetrahydrocannabinol (THC), causing psychoactive effects via activation of the CB1R, and the non-psychotropic Cannabidiol (CBD), with a higher affinity for the CB2R, as active components led step by step to the discovery of the endocannabinoid system (ECS). The ECS consist of endocannabinoids, cannabinoid receptors and enzymes involved in their biosynthesis, degradation and transport, as well as of various cell types and signalling pathways (Lu and Mackie 2021). In addition to mediating physiological processes, the ECS is also involved in various pathological processes of inflammation, neurodegeneration and cancer. Thus, therapeutic approaches addressing the ECS are of great interest. The best studied cannabinoid receptors so far are the G-protein coupled receptors (GPCRs) CB1R and CB2R sharing 44% overall sequence identity (Munro et al. 1993). The CB1R is highly expressed in neurons and glial cells in the brain and peripheral nervous system where it modulates various functions such as memory, cognition, emotion and pain control (Silvestri and Marzo 2013; Vendel and Lange 2014). CB2R is involved in regulating the immune system and is expressed at high levels in the spleen and at low levels in the brain (Latek et al. 2011; Du et al. 2023; Govaerts et al. 2004). In comparison with the psychotropic effects of CB1R agonists and the severe side effects of CB1R antagonists in humans, CB2R ligands are regarded as safe (Franco et al. 2022). The CB2R is upregulated in neurodegenerative and neuroinflammatory disorders, such as Huntington's, Alzheimer's and Parkinson's diseases, as well as various cancers and represents a promising therapeutic target (Fernández-Ruiz et al. 2015; Benito et al. 2003; Jia et al. 2014; Roche and Finn 2010; Blasco-Benito et al. 2019; Ellert-Miklaszewska et al. 2007). Anti-inflammatory effects of the CB2R are mediated by orthosteric agonists and allosteric modulators (Navarro et al. 2021). Natural CBR-ligands such as the phytocannabinoid THC (*K*_i(CB1R)_ = 25 nM, *K*_i(CB2R)_ = 36 nM), the endocannabinoids anandamide (AEA; *K*_i(CB1R)_ = 240 nM, *K*_i(CB2R)_ = 440 nM) and 2-arachydonilglycerol (2-AG; *K*_i(CB1R)_ = 3423 nM, *K*_i(CB2R)_ = 1194 nM) possess low affinity and CB1R/CB2R-selectivity compared to the synthetic agonists and antagonists developed in the last decades, with *K*_i_ values in the low to subnanomolar range (McPartland et al. 2007; Kosar et al. 2024) and some of them are currently being investigated in clinical studies. Actually, the first clinical trials with CB2R selective agonists, such as NTRX-07 in healthy volunteers (Phase 1, NCT04375436), JBT-101 for cystic fibrosis and systemic lupus erythematosus (Phase 2, NCT03451045, NCT03093402) and CB2R antagonists, such as TT-816 in combination with a Programmed cell death protein 1 (PD-1) inhibitor for different cancer entities (Phase 1/2, NCT05525455) are being conducted (Kosar et al. 2024).

Hence, the need for individualized diagnosis and therapeutic monitoring of CB2R expression in various diseases of the central nervous system, such as neuroinflammation, neurodegeneration, and glioma relies on sensitive methods, such as non-invasive molecular imaging with positron emission tomography (PET) (Cools et al. 2023). In the last decades, several ^11^C and ^18^F-labeled CB2R ligands have been developed (Hou et al. 2021; Ni et al. 2021). However, the majority of radioligands developed for the assessment of the CB2R over the last decade failed due to a lack of selectivity, binding affinity, ability to sufficiently cross the BBB and insufficient metabolic stability in vivo. In a recent approach, a CB2R-positive allosteric modulator (CB2R PAM) was developed to avoid inherent side effects of orthosteric ligands, whereby the CB2R PAM increased and stabilized the binding of the non-selective cannabinoid receptor agonist [^3^H]CP55,940 to CB1R and CB2R (Gado et al. 2019). In contrast, CBD was recently characterised as a CB2R-negative allosteric modulator (Franco et al. 2022). Furthermore, the first bitopic orthosteric/allosteric ligands showed promising results regarding the improvement of receptor selectivity and affinity (Ferrisi et al. 2023; Gado et al. 2022). However, to the best of our knowledge, two CB2R radiotracers have been evaluated by first-in-human studies so far, [^11^C]NE40 (Ahmad et al. 2013) and [^11^C]MDTC (Du et al. 2023) and currently a clinical phase I trial (NCT05880563) is on the way to investigate the biodistribution of [^18^F]RoSMA-18-*d*_6_. An ^18^F-labeled thiazole-based derivative for CB2R PET imaging, [^18^F]JHU94620 was developed by our group which revealed excellent in vitro affinity and selectivity (*K*_i(hCB1R)_ = 380 nM, *K*_i(hCB2R)_ = 0.4 nM), specific binding to the CB2R in the mouse spleen, as well as an increased uptake in the brain of systemically LPS-treated mice (Moldovan et al. 2016). However, [^18^F]JHU94620 was characterized by insufficient metabolic stability leading to a parent fraction of 0.07 in CD-1 mouse plasma and 0.36 in brain at 30 min post-injection (p.i.). The high proportion of radiometabolites in the brain results in a high signal-to-background ratio and a biased quantification of the radiotracer that is specifically bound to the CB2 receptor. High-performance liquid chromatography (HPLC) analysis of mouse brain samples revealed the presence of hydrophilic radioactive metabolites probably formed by the degradation of the *N*-fluorobutyl chain. To test this hypothesis, we decided to develop deuterated isotopologues of [^18^F]JHU94620. The use of deuterium to improve the metabolic stability was previously demonstrated for radiotracers such as [^18^F]FLUDA (Lai et al. 2021), [^18^F]RoSMA-18-*d*_6_ (Haider et al. 2020) and other small molecules (Martino et al. 2023; Kuchar and Mamat 2015). As the pertinence of using deuterated [^18^F]*N*-fluorobutyl residues has not been hitherto evaluated in vivo, the degree of deuteration required to improve metabolic stability at this part of the molecule remained to be assessed. Therefore, two derivatives were developed, namely [^18^F]JHU94620-*d*_4_ bearing the deuterium atoms at the *N*-fluorobutyl flank methylene groups, and [^18^F]JHU94620-*d*_8_ bearing a fully deuterated *N*-fluorobutyl chain.

After the evaluation of the metabolic stability of both [^18^F]JHU94620-*d*_4_ and [^18^F]JHU94620-*d*_8_, we performed an intensive biological evaluation of [^18^F]JHU94620-*d*_8_, showing the higher metabolic stability, consisting of in vitro binding studies and dynamic PET studies in healthy rats and rats with an overexpression of the functional inactive *h*CB2R(D80N) protein in the striatal region.

## Materials and methods

### Organic chemistry

All chemicals and reagents were purchased from commercial sources and used without further purification. Moisture-sensitive reactions were conducted under an argon atmosphere with oven-dried glassware and anhydrous solvents. Reaction progress was monitored by thin-layer chromatography (TLC) using Alugram® SIL G/UV_254_ pre-coated plates (Macherey–Nagel, Düren, Germany). The spots were identified by using a UV lamp or by dipping the plates into a potassium permanganate solution (3 g KMnO_4_, 20 g K_2_CO_3_, 0.25 mL glacial acid, 300 mL water). For purification of products flash column chromatography was used with silica gel 40–63 μm (VWR International Chemicals, Darmstadt, Germany). ^1^H-, ^13^C- and ^19^F-NMR spectra were recorded on VARIAN Mercury plus (300 MHz for ^1^H-NMR, 75 MHz for ^13^C-NMR, 282 MHz for ^19^F-NMR) and BRUKER DRX-400 (400 MHz for ^1^H-NMR, 100 MHz for ^13^C-NMR, 377 MHz for ^19^F-NMR), chemical shifts (δ) in parts per million (ppm) are related to internal tetramethylsilane and coupling constants (*J*) are given with 0.1 Hz. High-resolution mass spectra (HRFT-MS) were recorded on a FT-ICR APEX II spectrometer (Bruker Daltonics, Bruker Corporation, Billerica, USA) using electrospray ionization (ESI). The purity of all the tested compounds was ≥ 95% as determined by HPLC [Jasco, MD-2010Plus, LG-2080-04S, DG-2080-54, AS-2055Plus, LC-NetII/ADC, λ = 280 nm, column ReproSil-Pur Basic C18-HD (250 × 4.6 mm, 5 μm, Dr. Maisch GmbH, Ammerbruch, Germany), gradient MeCN/20mMAA from 10/90 to 90/10, to 10/90 (*v/v*) over 30 min, flowrate 1 mL/Min].

**Scheme 1:**
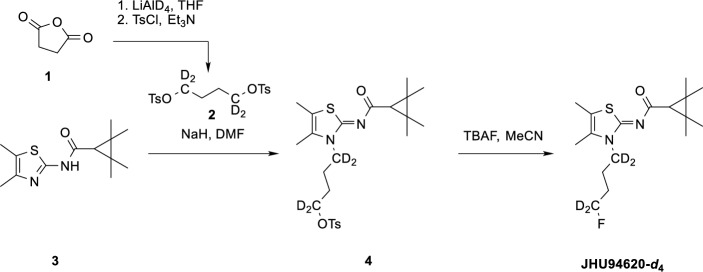
Synthesis of JHU94620-*d*_4_

(*Z*)-4-(4,5-Dimethyl-2-((2,2,3,3-tetramethylcyclopropane-1-carbonyl)imino)thiazol-3(2*H*)-yl)butyl-1,1,4,4-*d*_4_-4-methylbenzenesulfonate (**4**):

Compound **2** was synthesized as described in the literature (Colonna et al. 2011) by the reduction of succinic anhydride with LiAlD_4_ followed by double tosylation with TsCl in the presence of Et_3_N. To a solution of compound **2** (1 eq, 0.6 mmol) and compound **3** (1.5 eq, 0.9 mmol) in 3 mL DMF, NaH (60%, 2 eq, 1.2 mmol) was added and the mixture was heated to 60 °C for 1 h under argon atmosphere. The solvent was then removed under reduced pressure. The residue was taken up in ethyl acetate (EA) (10 mL) and washed with an aqueous 5% NaHCO_3_ solution (10 mL) and then with saturated aqueous NaCl solution (10 mL). Drying over MgSO_4_ and removal of the solvent gave a yellow oil which was purified by column chromatography (silica gel, EA: PE, 1/20 to 1/4). Compound **4** was obtained as a white solid with a yield of 33%. ^1^H-NMR (400 MHz, CDCl_3_) δ/ppm 7.81 (d, *J* = 8.3 Hz, 2H), 7.36 (d, *J* = 8.0 Hz, 2H), 2.47 (s, 3H), 2.16 (s, 6H), 1.75 (m, 4H) 1.50 (s, 1H), 1.33 (s, 6H), 1,21 (s, 6H). HRMS (ESI +): *m*/*z* (%) = 483.2278, calc. 483.2284 for C_24_H_31_D_4_FN_2_O_4_S_2_^+^ [M + H]^+^.

**Scheme 2:**
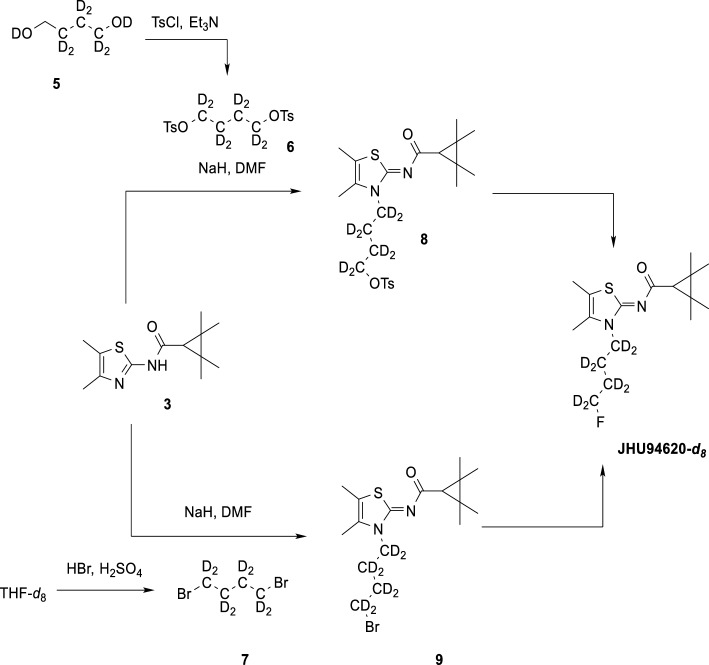
Synthesis of JHU94620-*d*_8_

(*Z*)-*N*-(3-(4-Fluorobutyl-1,1,4,4-*d*_4_)-4,5-dimethylthiazol-2(3*H*)-ylidene)-2,2,3,3-tetramethylcyclopropane-1-carboxamide (JHU94620-*d*_4_):

To a solution of compound **4** (1 eq, 0.5 mmol) in 3 mL THF was added TBAF (1M in THF, 2 eq, 1 mmol) and the mixture was heated to 50 °C for 1 h under argon atmosphere. The solvent was then removed on a rotary evaporator. The residue was taken up in ethyl acetate (10 mL) and washed with a 5% aqueous NaHCO_3_ solution (10 mL) and then with saturated NaCl solution (10 mL). Drying over MgSO_4_ and removal of the solvent gave a yellow oil which was purified by column chromatography (silica gel, EA: PE, 1/20 to 1/4). Compound JHU94620*-d*_4_ was obtained as a white solid with a yield of 72%.

^1^H-NMR (300 MHz, CDCl_3_) δ/ppm 2.20 (s, 3H), 2.18 (s, 3H), 1.55 (s, 1H), 1.36 (s, 6H), 1.24 (s, 6H). HRMS (ESI +): *m*/*z* (%) = 331.2152, calc. 331.2152 for C_17_H_24_D_4_FN_2_OS^+^ [M + H]^+^.

(*Z*)-4-(4,5-Dimethyl-2-((2,2,3,3-tetramethylcyclopropane-1-carbonyl)imino)thiazol-3(2*H*)-yl)butyl-1,1,2,2,3,3,4,4-*d*_8_-4-methylbenzenesulfonate (**8**):

Compound **6** was synthesized by double tosylation of the commercially available diol **5** with TsCl in the presence of Et_3_N. Compound **8** was synthesized by the same procedure as compound **4** .^1^H-NMR (400 MHz, CDCl_3_) δ/ppm 7.81 (d, *J* = 8.3 Hz, 2H), 7.36 (d, *J* = 8.0 Hz, 2H), 2.47 (s, 3H), 2.16 (s, 6H), 1.50 (s, 1H), 1.33 (s, 6H), 1.21 (s, 6H). HRMS (ESI +): *m*/*z* (%) = 487.2537, calc. 487.2535 for C_24_H_27_D_8_N_2_O_4_S_2_^+^ [M + H]^+^.

Compound **7** was synthesized starting from THF-*d*_8_ according to the literature (Kawamoto et al. 2015).

(*Z*)-*N*-(3-(4-Fluorobutyl-1,1,2,2,3,3,4,4-*d*_8_)-4,5-dimethylthiazol-2(3*H*)-ylidene)-2,2,3,3-tetramethylcyclopropane-1-carboxamide (JHU94620-*d*_8_):

JHU94620-*d*_8_ was synthesized by the same procedure as compound JHU94620-*d*_4_. ^1^H-NMR (300 MHz, CDCl_3_) δ/ppm d 2.20 (s, 3H), 2.18 (s, 3H), 1.93–1.81 (m, 2H), 1.77–1.68 (m, 2H), 1.55 (s, 1H), 1.36 (s, 6H), 1.24 (s, 6H). HRMS (ESI +): *m*/*z* (%) = 335.2402, calc. 335.2403 for C_17_H_20_D_8_FN_2_OS^+^ [M + H]^+^.

### Radiochemistry

**Scheme 3:**
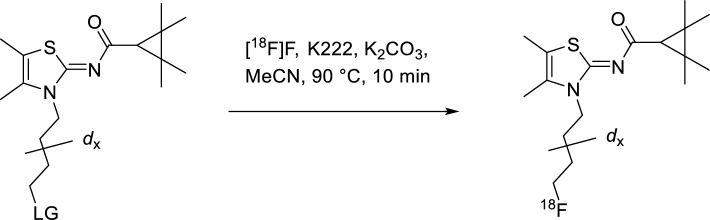
General synthesis scheme for [^18^F]JHU94620-*d*_x_; x = 0, 4 or 8

#### Quality control

Unless stated otherwise, radio-high performance liquid chromatography (radio-HPLC) analyses were performed on a JASCO LC-2000 system (JASCO Labor- und Datentechnik GmbH, Pfungstadt, Germany), incorporating a PU-2080Plus pump, AS-2055Plus auto-injector (100 µL sample loop), and a UV-2070Plus detector coupled with a γ-detector (GABI Star, Raytest Isotopenmessgeräte GmbH, Straubenhardt, Germany). A Reprosil-Pur 120 C18-AQ column (250 × 4.6 mm, 5 µm, Dr. Maisch HPLC GmbH, Ammerbuch, Germany), and a solvent system consisting of 10% MeCN/ 20 mM NH_4_OAc_aq_ and 90% MeCN/ 20 mM NH_4_OAc_aq_ was used with a flow rate of 1 mL/min and UV detection at 312 nm.

For **method A**, linear gradient elution: 0–10 min 10%, 10–25 min 10–90%, 25–35 min 90%, 35–40 min 90–10%, 40–45 min 10% MeCN/ 20 mM NH_4_OAc_aq_.

For **method B**, linear gradient elution: 0–5 min 10%, 5–18 min 10–90%, 18–25 min 90%, 25–26 min 90–10%, 26–36 min 10% MeCN/ 20 mM NH_4_OAc_aq_.

For **method C**, a JASCO X-LC system (JASCO Labor- und Datentechnik GmbH), a Poroshell 120 EC-C18 column (100 × 3 mm, 3.5 µm, Agilent Technologies, Santa Clara, CA, USA), and a solvent system consisting of 5% MeCN/ 20 mM NH_4_OAc_aq_ (overall volume) and 80% MeCN/ 20 mM NH_4_OAc_aq_ (overall volume) was used. Step gradient elution: 0–1.5 min 5%, 1.5–4 min 20–60%, 4–10 min 60–80%, 10–12 min 80%, 12–15 min 5%; flow rate: 0.7 mL/min.

For **method D**, isocratic elution, 65%MeCN/ 20 mM NH_4_OAc_aq_.

Radio-TLC was performed on Alugram® SIL G/UV_254_ pre-coated plates (Macherey–Nagel, Düren, Germany) with PE:EA (1:1, *v*/*v*). The plates were exposed to storage phosphor screens (BAS IP MS 2025 E, GE Healthcare Europe GmbH, Freiburg, Germany) and recorded using the Amersham Typhoon RGB Biomolecular Imager (GE Healthcare Life Sciences). Images were quantified with the ImageQuant TL8.1 software (GE Healthcare Life Sciences).

#### Manual radiosynthesis

The aqueous [^18^F]fluoride (1 to 2 GBq) obtained after irradiation was added to 1 mL water, fixed on an anion exchange cartridge (QMA) and eluted with a solution containing K_2_CO_3_ (50 µL, 20% aqueous), MeCN (1 mL), H_2_O (200 µL) and Kryptofix (K222, 5.6 mg). The azeotropic drying of the complex was microwave-assisted (power cycling, 75 W, 50–60 °C, argon flow). The formed [^18^F]F^−^/K222/K^+^-complex was mixed with 2 mg of precursor **4** or **8** (in 600 µL MeCN) and the reaction mixture was then stirred at 90 °C for 10 min. To determine the radiochemical conversion (RCC), an aliquot was taken and analysed by Radio-thin layer chromatography (radio-TLC; 49 ± 4%, n = 3) and radio-HPLC (48 ± 5%, n = 3). Purification and isolation of the radiotracer was performed by semi-preparative RP-HPLC (column: ReproSil-Pur 120 C18-AQ, 250 × 10 mm, 5 µm; eluent: 65% MeCN/20 mM NH_4_OAc (aq.); flow rate: 4.2 mL/min). The collected product fraction was diluted with water (20 mL), absorbed on a Sep-Pak® C18-Plus cartridge, washed with H_2_O (2 mL) and eluted with ethanol (EtOH) (1.0 mL). Subsequently, the solvent was removed by heating to 70 °C under a gentle nitrogen stream and formulated in 0.9% NaCl with ≤ 10% ethanol, *v/v*. The purity and identity of the product was confirmed by radio-HPLC (methods B and D) and radio-TLC. The product, [^18^F]JHU94620-*d*_*4*_ or -*d*_*8*_ was obtained within a synthesis time of approximately 102 min, with a RCY of 22 ± 2%, a RCP of > 99% and a molar activity (A_m_) of 200 ± 20 GBq/µmol (EOS, n = 3).

For quality control, the RCC and RCP were determined using radio-HPLC, method B, and A_m_ using method D. Radio-TLC was performed as described in the Quality control section.

#### *Automated radiosynthesis of [*^*18*^*F]JHU94620-d*_*x*_

Remotely-controlled radiosynthesis was performed using a Synchrom R&D EVO III automated synthesizer (Elysia-Raytest, Germany). Briefly, [^18^F]fluoride (4–6 GBq) was trapped on a Waters QMA cartridge and eluted with a solution containing K_2_CO_3_ (50 µL, 20% aqueous), MeCN (1 mL), H_2_O (200 µL) and Kryptofix (K222, 5.6 mg) into the reaction vessel and dried via azeotropic distillation and 1.5 mL of dried MeCN was added. After complete dryness, a solution containing 2 mg of precursor **4** or **8** in 800 µL MeCN was added, and the reaction mixture was stirred at 90 °C for 10 min. Upon cooling to 40 °C, the reaction mixture was diluted with 4 mL H_2_O, and the resulting solution was transferred to the semi-preparative HPLC. [^18^F]JHU94620-*d*_4_ or -*d*_8_ was collected in the HPLC collection vial containing 40 mL of H_2_O and trapped in the Sep-Pak® C18 light cartridge. The cartridge was washed with 2 mL H_2_O, and [^18^F]JHU94620-*d*_4_ or -*d*_8_ was eluted with 1.2 mL EtOH. This ethanolic solution was transferred outside of the shielded cell, the solvent was evaporated at 70 °C in a gentle stream of nitrogen for 5–10 min, and [^18^F]JHU94620-*d*_4_ or -*d*_8_ was diluted with 0.9% NaCl aqueous solution to a final proportion of 10% EtOH/NaCl_aq _for further biological characterization. The total synthesis time was about 70 min, RCY of 20 ± 6% and the molar activity (A_m_) was 220 ± 35 GBq/µmol (EOS; n = 10).

The radiosynthesis of [^18^F]JHU94620-*d*_8_ starting from precursor **9** was performed as described by Moldovan et al. (Moldovan et al. 2016).

Quality control was performed as described before for the “Manual synthesis”.

### In vitro* metabolism*

#### Metabolic degradation by liver microsomes

For microsomal incubations liver microsomes from human (HLM, 50 donors, 20 mg/mL) and rat (RLM, Sprague Dawley, 20 mg/mL) were purchased from Thermo Fisher Scientific GmbH (Dreieich, Germany). Testosterone and β-nicotinamide adenine dinucleotide 2′-phosphate reduced tetrasodium salt (NADPH) were purchased from Sigma-Aldrich (Merck KGaA, Darmstadt, Germany) and Dulbecco’s PBS (DPBS) (without Ca^2+^ and Mg^2+^) was purchased from Biochrom GmbH (Berlin, Germany). Analyses of samples for comparison of radiometabolite patterns were performed by radio-HPLC using method B, for determination of time dependency of the metabolic depletion using method C.

All incubations had a final volume of 250 µL and were performed in PBS (pH 7.4) according to a previously described procedure (Ludwig et al. 2019). The final concentrations are given in brackets. HLM or RLM were diluted in PBS (1 mg/mL each) and approx. 8 MBq [^18^F]JHU94620-*d*_8_ (no-carrier-added, A_m_ = 169 GBq/µmol, ~ 0.2 µM; EtOH 0.2%) in 25 µL PBS were added, vigorously mixed and kept on ice. After pre-incubation at 37 °C for 5 min a freshly prepared and equally treated solution of NADPH (2 mM) was added. The mixtures were vortexed and shaken gently at 37 °C using the BioShake iQ (QUANTIFOIL Instruments, Jena, Germany) for 5, 10, 15, 30, 45, and 60 min (for HLM) or 15, 30, 45, and 60 min (for RLM). The incubations were terminated by the addition of a 250 µL MeOH/H_2_O mixture (9:1, *v/v*, − 20 °C). After vigorous shaking for 30 s, the mixtures were kept on ice for 15 min and centrifuged at 11,000 g for 10 min. The supernatants were separated and analyzed by radio-HPLC to determine the fractions of unchanged [^18^F]JHU94620-*d*_8_. Efficiencies of extracted activity were determined as described in “Quantification of radiometabolites”. For negative controls, incubations contained no microsomes, no NADPH or neither both of them. As positive control testosterone was used as substrate instead of [^18^F]JHU94620-*d*_8_ and the subsequent HPLC analysis (method C) was performed with UV detection at 245 nm.

To determine the metabolic stability in HLM and RLM, both the natural logarithm of the determined unchanged percentage of [^18^F]JHU94620-*d*_8_ was plotted versus incubation time, which allowed the calculation of the in vitro half-lives as described previously (Chai et al. 2022).

#### Generation of radiometabolites for autoradiographic studies

To obtain samples for autoradiographic studies, [^18^F]JHU94620-*d*_8_ (~ 10 MBq) was incubated as described above in Sect. (Metabolic degradation by liver microsomes) for 60 min with HLM: a) in the presence of NADPH to obtain complete conversion and b) without NADPH as a control containing only the unchanged radiotracer. The supernatants obtained after MeOH addition and centrifugation were concentrated to a residual volume of 250 µL.

### Binding studies

For binding studies crude homogenates of CHO(*h*CB2R) cells (obtained from Paul L. Prather, Department of Pharmacology and Toxicology, College of Medicine, University of Arkansas for Medical Sciences, USA) were incubated with the reference compound (self-blocking) or the CB2R-specific partial agonist GW405833 (Tocris Bioscience, Bristol, UK) with indicated concentrations at room temperature for 90 min. The total volume of 1 mL of each sample consisted of 600 µL incubation buffer (50 mM Tris HCl pH7.4, 5 mM MgCl_2_, 1 mM EDTA and 1% Bovine Serum Albumin), 200 µL cell membrane homogenates, 100 µL competitor or binding buffer (total binding) and 100 µl of binding buffer with [^18^F]JHU94620-*d*_8_ (about 100 kBq per vial, corresponding to 0.8 to 1.6 nM). Incubation was terminated by rapid filtration through a GF-B glass fibre filter, pre-treated with 0.5% PVP with 0.1% Tween 20 solution, using a 48-well cell harvester, washed six times with buffer and filter bound activity was subsequently measured in a γ-counter (1480 WIZARD, Perkin Elmer, Turku, Finland).

### Animals

#### Ex vivo* radiometabolite analysis*

Female adult CD-1 mice (25–42 g body weight) were used for radiometabolite analysis of [^18^F]JHU94620-*d*_4_ (n = 2, 33 and 34 MBq) and [^18^F]JHU94620-*d*_8_ (n = 3, 26–33 MBq) at 30 min after intravenous tail vein injection of the radiotracer to allow for the comparison of radiometabolite analysis of [^18^F]JHU94620 (Moldovan et al. 2016). The mice were immobilized by short isoflurane narcosis for the injection of the radiotracer in a restrainer and awakened mice were kept in an isolated cage until euthanisation by cervical dislocation under isoflurane narcosis. Male Wistar rats (n = 12, 613–794 g) were used for radiometabolite analysis of [^18^F]JHU94620-*d*_8_ at different time points (5, 15, 30 and 60 min p.i.) after intravenous tail vein injection of the radiotracer (37–74 MBq) for the correction of the input function in pharmacokinetic analysis. Rats were kept under isoflurane narcosis (gas mixture: 2.5% isoflurane and O_2_) on a warmed plate until euthanization by decapitation of deep narcotized animals. Plasma samples were obtained by centrifugation with 5500 g of blood samples for 2 min at 4 °C. Resected tissues brain and spleen were kept on ice and minced for 3 × 20 s with adding 0.5 to 1 mL of 0.9% NaCl using a Precellys lysing kit and Minilys homogenizer (Bertin Technologies SAS, Montigny-le-Bretonneux, France).

#### Quantification of radiometabolites

The samples were further processed for subsequent radio-chromatographic analyses. Two consecutive extractions were performed in duplicates for plasma and brain determinations. Plasma, brain and spleen samples were added to the four time the volume of an ice-cold MeOH/H_2_O mixture (9:1, *v/v*). The samples were vortexed for 3 min, incubated on ice for 5 min and centrifuged at 8,600 g for 5 min. Supernatants were collected and the precipitates were re-dissolved in 100 µL of extraction solvent and the extraction procedure was repeated. The activities of supernatants and precipitates were measured in a γ-counter (1480 WIZARD, Perkin Elmer), and the extraction efficiencies were calculated as the ratio of radioactivity in the supernatant to the radioactivity in the original sample (supernatant + precipitate). The supernatants from both extractions were combined, concentrated at 70 °C under argon stream up to a remaining volume of 100 µL, and subsequently analysed by analytical radio-HPLC (method A or B).

#### Quantitative autoradiography

For quantitative autoradiography in different species, 10 µm cryosections from mouse, rat and piglet spleen were thawed, dried, and pre-incubated with incubation buffer for 10 min, dried under an airstream. Subsequently, the incubation buffer containing [^18^F]JHU94620-*d*_8_ (59–137 GBq µmol^−1^, 1.7–3.6 nM) was added alone or together with indicated concentrations of the reference compound. Additionally, to assess the non-specific binding of [^18^F]JHU94620-*d*_8_ to cryosections GW405833 was added instead of the reference compound. After 90 min, cryosections were washed two times for 2 min with ice-cold 50 mM Tris HCl, pH7.4 and 10 s in ddH_2_O and subsequently dried under an airstream. For the calculation of the radioactivity concentration, 5 µl of five serial 1:2 dilutions of the incubation buffer with [^18^F]JHU94620-*d*_8_ were air-dried on a microscopic slide. For binding studies in rat spleen cryosections by autoradiography with HLM-derived radiometabolites of [^18^F]JHU94620-*d*_8_ obtained from the radiometabolite assay, the samples were added to the incubation buffer to a final activity concentration of 0.2 MBq mL^−1^ and protocol was followed as described above. Subsequently, the slides were exposed to a phosphor imager plate. After scanning at high sensitivity and a 12.5 µm resolution with a CR35 Bio image plate scanner (Raytest Isotopenmessgeräte GmbH, Straubenhardt, Germany). The analyses of the autoradiographic images were performed with the AIDA 5.1 software (Elysia-Raytest, Angleur, Belgium) resulting in a resolution of 12.617 pixel × 12.616 pixel per µm^2^ (1 voxel = 1592 µm^3^ ≙ 1.59 10^–3^ µg wet tissue) and inhibition curves were created with GraphPad Prism 9.5.1 (GraphPad Inc., La Jolla, CA).

The *K*_D_ values were calculated with GraphPad Prism according to the modified Cheng-Prusoff method for homologous competitive binding studies, where the *K*_D_ = *K*_i_ = IC50—[radioligand] and the *B*_max_ = specific binding / [radioligand] / ((K_D_ + [radioligand])) (Motulsky and Neubig 2000), assuming a total protein content of 8.2% (1.3 10^–4^ µg protein per voxel) in the spleen (Zaia et al. 2000) or by determining the protein concentration of the cell membrane lysates using a BCA assay (Thermo Scientific, Braunschweig, Germany). The fractional occupancy defined as occupied binding sites per total binding sites is used to describe the binding fraction specifically bound to CB2R compared to unspecific binding, where [L] is the concentration of the ligand at the start of incubation.

#### Dynamic PET imaging in rats

The dynamic biodistribution of the radiotracer in the lower body of rats was assessed by small animal PET (Nanoscan, Mediso, Hungary) over 60 min with a subsequently T1-weighted MR. Anaesthetized (2% isoflurane, carrier gas mixture of 40% air and 60% O_2_) male Wistar rats (body weight: 205–308 g, age: 8–10 weeks, no gender-specific differences were taken into account) were kept during imaging on a heated animal bed to sustain body temperature and were pretreated with vehicle (DMSO:Kolliphore EL:0.9% saline, 1:2:7), JHU94620-*d*_8_ (1.5 mg kg^−1^ bodyweight) or with GW405833 (5 mg kg^−1^ bodyweight) 5 min prior tracer application ([^18^F]JHU94620-*d*_8_: 7.7–28.2 MBq, 0.4 to 3.2 nmol kg^−1^ bodyweight), whereby all injections were administered intravenously. For the evaluation of the *h*CB2R-specific binding of the radiotracer in the brain, female Wistar rats (n = 6, 253–269 g, age: 8 month) were used carrying stereotactically injected constructs of AAV2/7-CaMKII0.4-intron-*h*CB2R(D80N) in the right striatum and AAV2/7-CaMKII0.4-intron-3flag-eGFP in the control/contralateral striatum (Vandeputte et al. 2011). 21–26 MBq corresponding to 0.2 to 2.0 nmol kg^−1^ bodyweight radiotracer were injected, whereas in displacement studies 5 mg kg^−1^ bodyweight GW405833 was administered 20 min after the radiotracer (each n = 3). The PET scans were performed six months after the stereotactic injections of the AAV2/7-constructs.

The acquisitions were performed in normal mode and a coincidence mode 1–5. For subsequent dynamic reconstructions list mode data were sorted into sinograms (12 × 10 s, 6 × 30 s, 5 × 60 s and 10 × 300 s). The frames were reconstructed by Ordered Subset Expectation Maximization applied to 3D (OSEM3D) sinograms corrected for decay and attenuation (4 iterations, 6 subsets and a voxel size of 0.4 mm^3^) with the Nucline software v2.01 (Mediso, Budapest, Hungary). Analyses of reconstructed studies were performed with PMOD software (v4.205, PMOD Technologies LLC, Fällanden, Switzerland) and results are expressed in standardized uptake values (SUVs) or normalized to a reference region (SUVr).

#### Pharmacokinetic modelling

From the wildtype rats used for in vivo metabolism studies a hematocrit content of 0.56 ± 0.04 (n = 11) was determined by a 5 min centrifugation of 50 µl blood in a hematocrit centrifuge at 500 g. The free plasma fraction (*f*_p_) of [^18^F]JHU94620-*d*_8_ was determined gravimetrically by ultrafiltration of 100 µl rat plasma samples incubated for 5, 15, 30 and 60 min at 37 °C for 20 min at 14,000 g utilizing Nanosep® centrifugal filters (10 kDa, Pall Laboratory, USA). The brain PET images were co-registered to the PMOD integrated Px Rat (W. Schiffer) T2 MR template and the brain stem, cerebellum, left cortex and left midbrain region from the corresponding brain atlas were used for the determination of the regional TACs. Due to high spillover signals from the *h*CB2R(D80N) expressing region not all provided atlas regions could be evaluated. Subsequently, the total tissue distribution (*V*_T_) was estimated by 2-tissue compartment modelling. For correction of the TACs, an averaged image-derived input function (pIDIF, plasma-to-whole blood ratio corrected), determined in biodistribution studies and the parent fraction derived from metabolism studies in Wistar rats (Watabe fit) and pIDIF (3-exponential fit) were integrated into the modelling process. The binding potential was estimated by a simplified reference tissue modelling (SRTM) using the cerebellar region as reference. The parametric maps were generated with the PXMOD module.

## Results

### Synthesis of the reference compound, radiosynthesis and metabolism studies

**Fig. 1:**
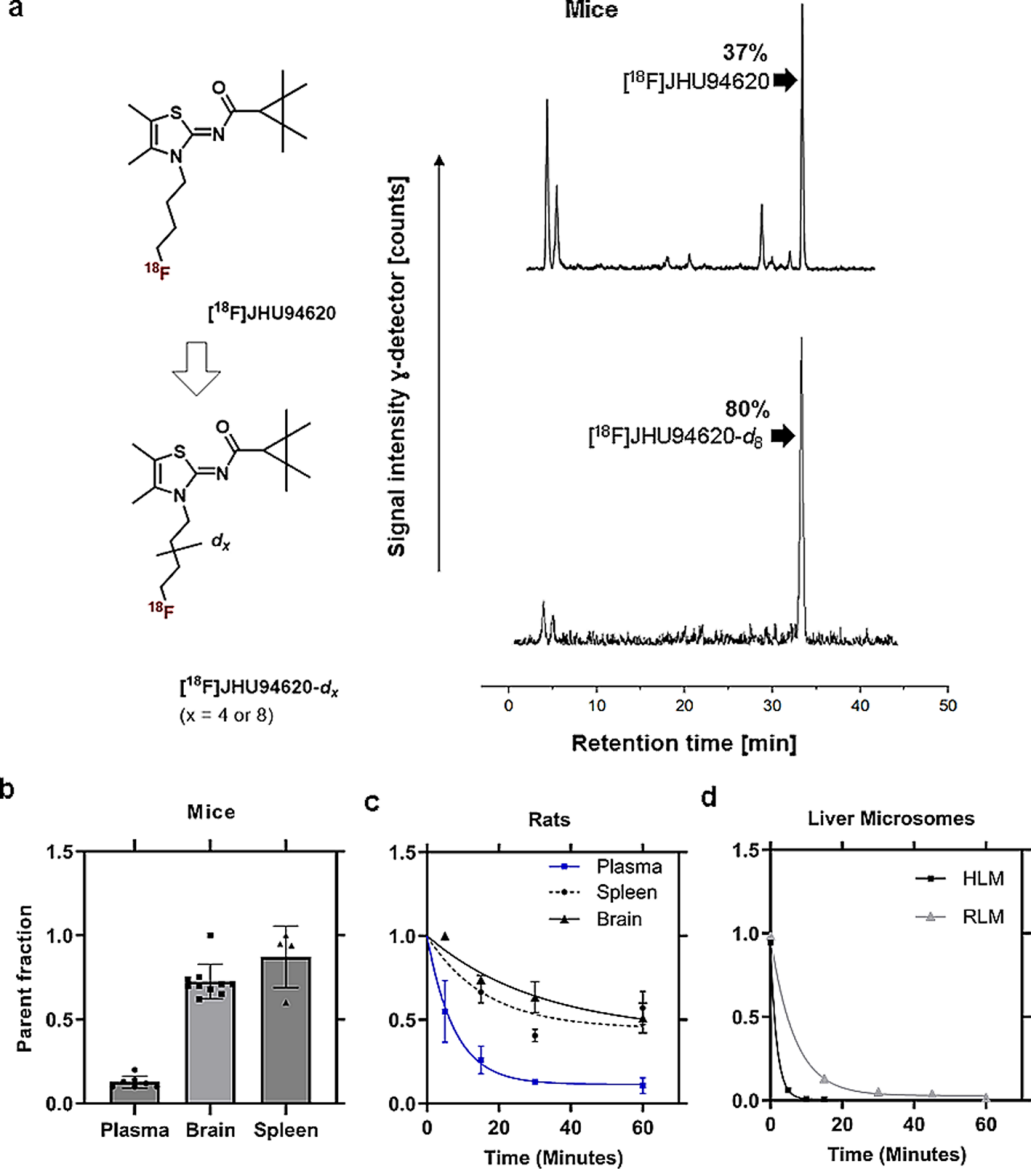
Development of [^18^F]JHU94620-*d*_8_ as well as its radiometabolite analysis in vivo in rodents and in vitro using liver microsome assays. **a** The structure of [^18^F]JHU94620, its deuterated analogues and representative radio-HPLC chromatograms (method A) of [^18^F]JHU94620 (upper HPLC chromatogram) and [^18^F]JHU94620-*d*_8_ (lower HPLC chromatogram) obtained from the brain of CD-1 mice 30 min p.i.; **b** parent fraction of the radiotracer determined in plasma, brain and spleen in CD-1 mice at 30 min (n = 7, 10 and 4, respectively, mean ± SD); **c** in Wistar rats over time (n = 3, each timepoint; mean ± SD); and **d** derived from human liver microsome (HLM, n = 1) and rat liver microsome assay (RLM, n = 1) assay, where the curves represent the exponential fits (**c** and **d**)

For the synthesis of [^18^F]JHU94620-*d*_*4*_, the LiAlD_4_-mediated reduction of succinic anhydride was used to introduce the deuterium atoms at positions 1 and 4 of the butyl chain. Compound [^18^F]JHU94620-*d*_8_ was synthesized by using 1,4-dibromobutane-*d*_8_ obtained by the bromination of THF-*d*_8_ with the HBr/H_2_SO_4_ system (Schemes 1, 2 and 3, Methods). Both, [^18^F]JHU94620-*d*_4_ and [^18^F]JHU94620-*d*_8_ were radiosynthesized similarly to our previous reports and evaluated regarding the metabolic stability in CD-1 mice at 30 min p.i. as shown in Fig. 1a and Figure S1. Both deuterated isotopologues have higher in vivo metabolic stability compared to [^18^F]JHU94620 with [^18^F]JHU94620-*d*_8_ being slightly superior to [^18^F]JHU94620-*d*_4_. The determined parent fraction of [^18^F]JHU94620-*d*_8_ in the brain of mice was 0.72 ± 0.05 and thus 50% higher compared to the non-deuterated isotopologue, whereas a parent fraction of 0.87 ± 0.10 in spleen and 0.13 ± 0.04 in blood plasma was determined (Fig. 1b and S1). Additionally, we investigated the metabolic stability of [^18^F]JHU94620-*d*_8_ at different time points in rats (Figs. 1c and S2). Hereby, the metabolic half-lives (t_1/2_) of the radiotracer of 7.6, 53.8 and 37.3 min, in plasma, brain and spleen, respectively, were estimated (Fig. 1c), whereas the HPLC radiometabolite pattern in rats was comparable to that in mice (Figures S1 and S2). As shown in the plasma-brain radiometabolite plot (Figure S3), the positive correlation of the radiometabolite concentration between the brain and plasma (R^2^ = 0.77) and the average slope of 0.89 indicate a higher radiometabolite fraction in plasma compared to the brain. Taken together, an accumulation of radiometabolites in the brain is unlikely.

In a further step, the phase I metabolism of [^18^F]JHU94620-*d*_8_ was investigated in vitro using RLM and HLM in the presence of NADPH (Jia and Liu 2007). The results, shown in Fig. 1d, S4 to S6, indicate a rapid metabolic degradation, which was more pronounced in HLM (t_1/2_ = 2.1 ± 0.7 min) than in RLM (t_1/2_ = 10.8 ± 2.4 min). Interestingly, the very hydrophilic radiometabolites (t_R_ ≤ 5 min), detected in plasma extracts of rodents (Figs. 1a and S4), were not found in the corresponding microsomal extracts (Figures S4 and S5). This finding suggests the possibility that these compounds are phase II radiometabolites.

### In vitro* binding affinity and autoradiography*

**Fig. 2:**
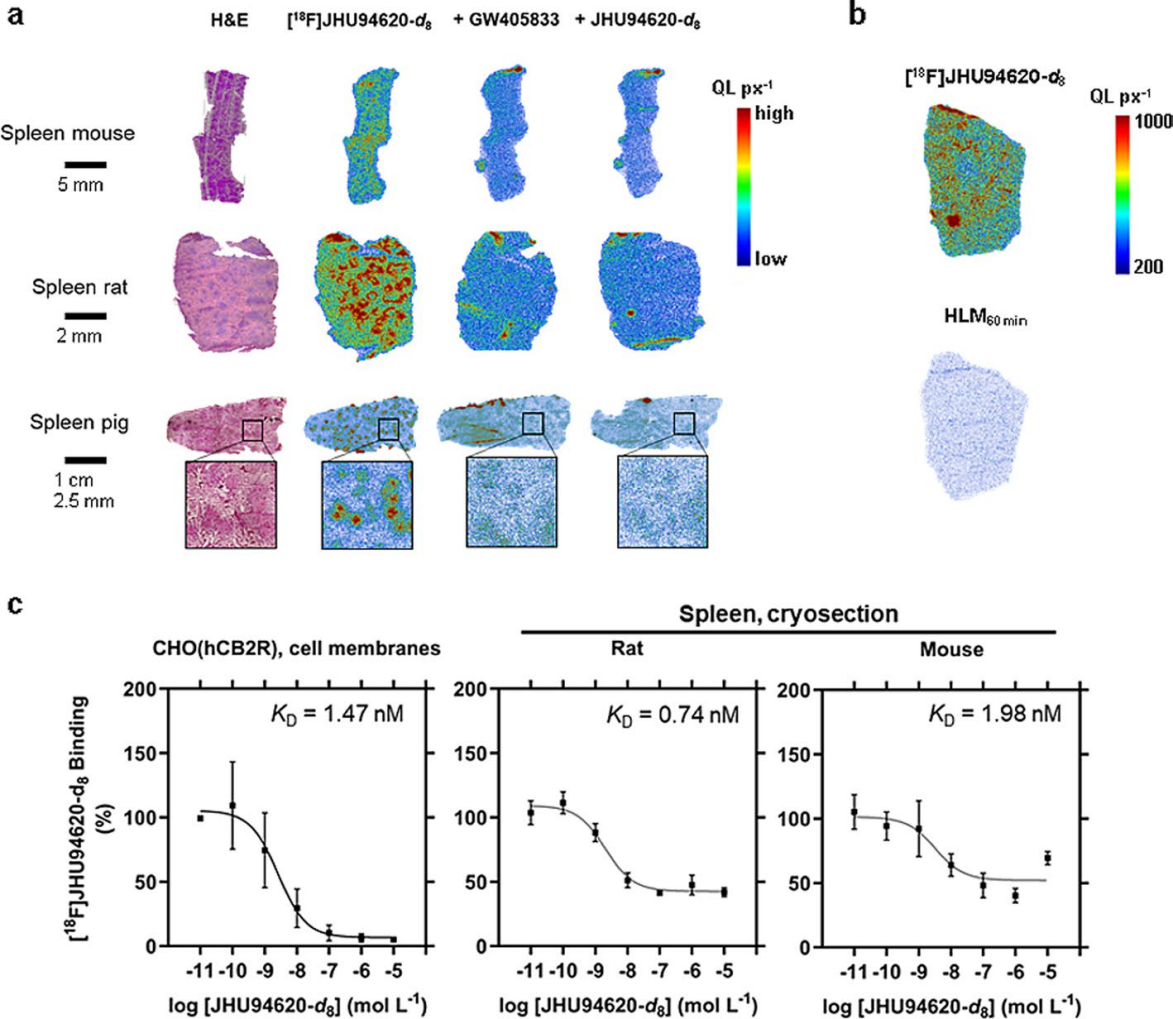
In vitro binding studies of [^18^F]JHU94620-*d*_8_ to the mice, rat, pig and human CB2R. **a** haematoxylin and eosin staining (H&E) of cryosections of mouse, rat and pig spleen, and autoradiograms provided in quantilumen per pixel (QL px^−1^) of total binding and blocking with 10 µM of GW405833 or self-blocking. Additionally, a magnified section of the pig spleen (square) is shown; **b** exemplary autoradiograms of rat spleen cryosections incubated with the radioligand or—metabolites derived from a 60 min human liver microsomal digestion (HLM_60min_); and **c** determination of the equilibrium dissociation constant (*K*_D_) by homologous displacement studies using cell membranes of CHO-cells overexpressing the human CB2R (*h*CB2R) or cryosections of rat and mouse spleens in autoradiography studies (means ± SD, n = 3)

To determine the specific binding of [^18^F]JHU94620-*d*_8_ we performed autoradiography on spleen cryosections from different species using a CB2R-specific agonist (GW405833 or SR144528), reference compound, PAM or a CB1R-specific agonist (SR141716A) for blocking studies and determined the *K*_D_ by homologous competition assays (Figs. 2 and S7 to S9).

A specific binding of [^18^F]JHU94620-*d*_8_ of 48 ± 6, 40 ± 18 and 22 ± 11% to CB2R could be demonstrated for rat, mouse, and pig spleen, respectively (Figs. 2a, S7). The observed heterogeneous binding intensities in the autoradiograms of the spleen cryosections could be explained by a higher CB2R receptor density in the white pulps. Additionally, we could not prove a CB2R-specific binding of the in vitro generated radiometabolites (7% compared to [^18^F]JHU94620-*d*_8_) derived from HLM metabolization assay in rat spleen cryosections (Figs. 2b and S8). The *K*_D_ values obtained from homologous competition assays using membrane fractions from CHO(*h*CB2R) and *K*_D_ values (0.74 to 1.98 nM) determined by autoradiography in rat and mouse spleen are in the same range, suggesting a low to moderate species-dependent CB2R affinity of [^18^F]JHU94620-*d*_8_ (Fig. 2c). The obtained *B*_max_ values were 2.7 pmol mg^−1^ protein in CHO(*h*CB2R) cell membrane fractions and 0.08 pmol mg^−1^ protein in the spleen of rats and mice. The co-incubation with 100 nM CB2R PAM did not increase the binding of [^18^F]JHU94620-*d*_8_ to CHO(*h*CB2R) cell membranes and rat spleen, however a weak competitive binding of PAM could be detected (Figure S9), hence close or same binding sites of the radioligand and CB2R PAM can be assumed.

### In vivo* uptake into the spleen and other abdominal tissues*

**Fig. 3:**
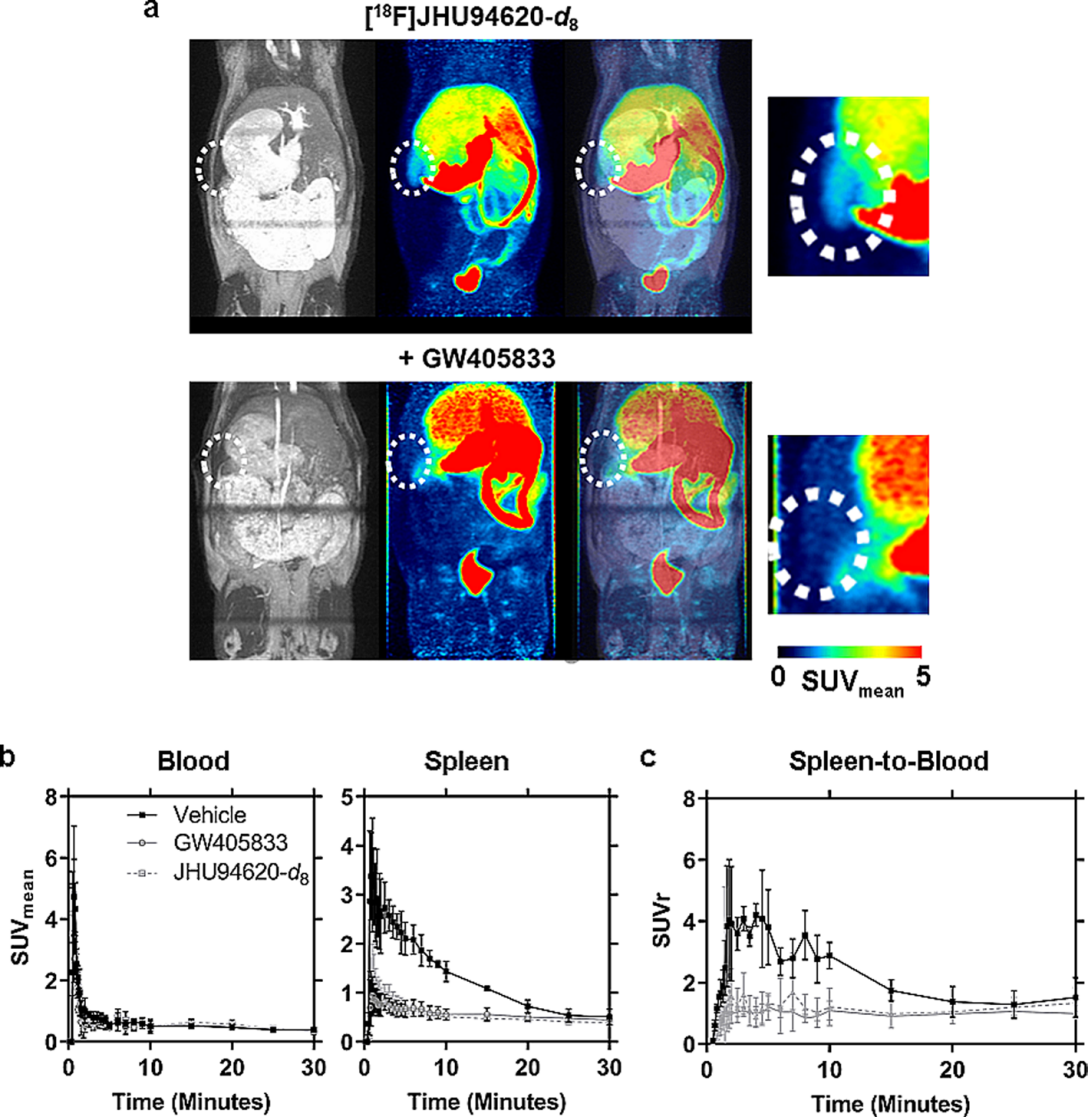
Dynamic PET study to investigate the CB2R-specific uptake of [^18^F]JHU94620-*d*_8_ into the spleen of rat over time. **a** Exemplary maximal intensity projections (MIPs) of T1-weighted MR, averaged PET frames from 0 to 30 min after administration of the radioligand under baseline and pre-blocking with GW405833, and merged MIPs (left to right), whereby the dotted circle marks the region of the spleen (magnified region in PET at the right); **b** Time activity curves of the mean standardized uptake value (SUV_mean_ ± SD) of the left ventricle (blood) and spleen; and **c** the SUV ratio (SUVr ± SD) of the spleen normalized to blood under baseline (Vehicle) and after pre-blocking with 1.5 mg kg^−1^ bodyweight GW405833 or JHU94620-*d*_8_ (n = 3)

To investigate the general biodistribution of [^18^F]JHU94620-*d*_8_ and to confirm the CB2R-specific binding of this radiotracer in the spleen in vivo we performed dynamic PET studies in healthy rats (Figs. 3 and S10).

The TACs of muscle, bone and lung showed the lowest uptake up to 30 min p.i.. We observed a fast clearance from the blood compartment by a mainly hepato-biliary excretion of radioactivity into the intestine and stomach, as well as a partial renal clearance most likely of the very hydrophilic radiometabolites (t_R_ ≤ 5 min, Figs. 3a and S10).

The blood TACs obtained after i.v. injection of GW405833 or the reference compound prior to [^18^F]JHU94620-*d*_8_ were comparable to those obtained in baseline conditions (1-way ANOVA, *p*-value > 0.05, Fig. 3b). However, the uptake of [^18^F]JHU94620-*d*_8_ into the spleen was significantly blocked between 2.5 and 20 min p.i. by both compounds (1-way ANOVA, *p*-value < 0.05, Fig. 3b), and the spleen-to-blood normalized TAC confirmed a reversible CB2R-specific binding of the radiotracer in vivo (Fig. 3c).

### hCB2R-specific binding in brain

**Fig. 4:**
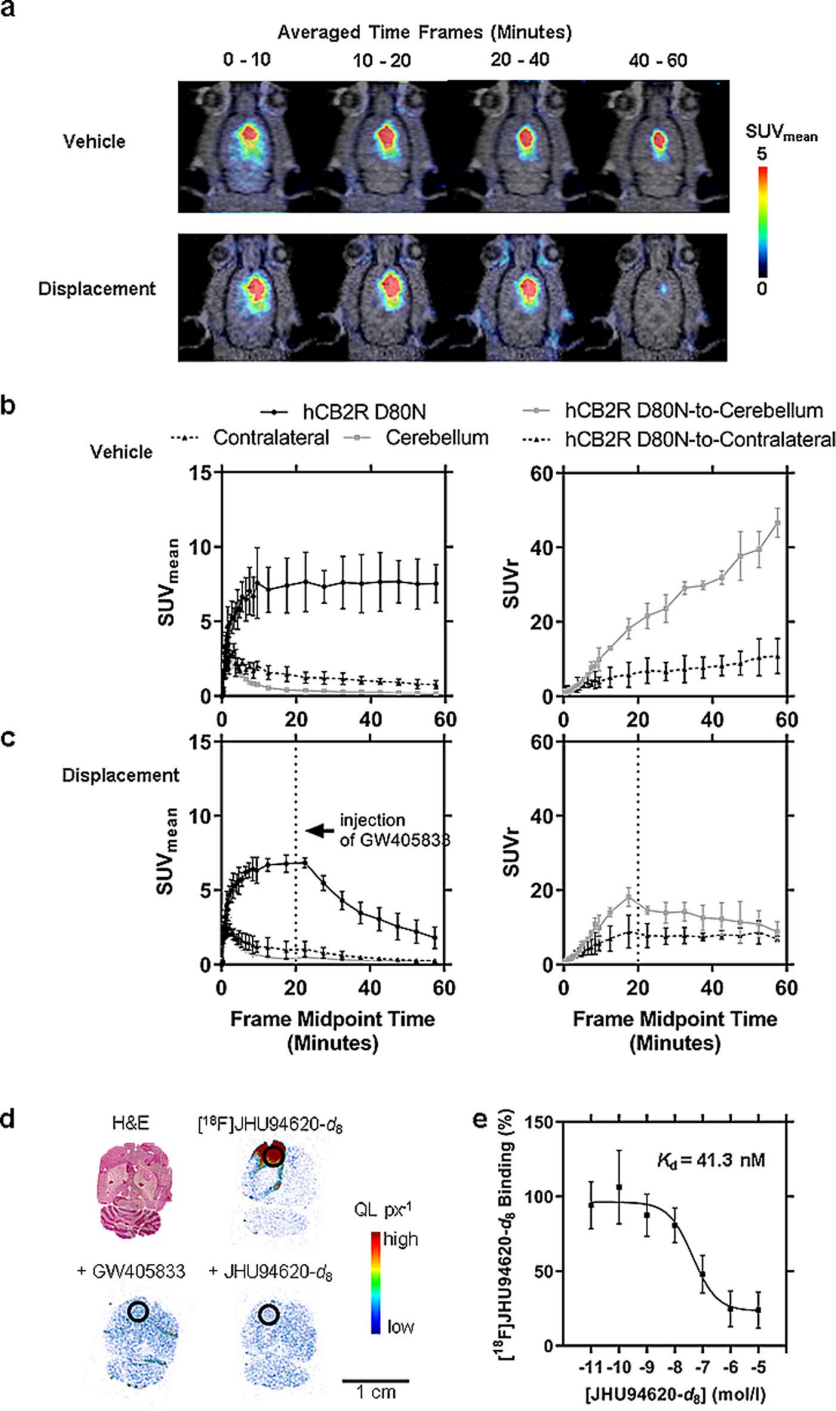
PET imaging of [^18^F]JHU94620-*d*_8_ in a rat model with a local *h*CB2R overexpression in the right hemisphere of the brain. **a** Representative coronal planes of merged MR and PET images of averaged time frames of the control group and displacement group (5mg kg^−1^ GW405833 injected 20 min after the start of the PET acquisition); **b** and **c** showing the corresponding time-activity curves (TACs) of the right (target region, *h*CB2R D80N) and the reference regions (contralateral and cerebellar region) expressed in mean standardized uptake values (SUV_mean_), as well as the normalized TACs to the reference regions (SUVr); **d** HE-staining and autoradiograms (in vitro) of a 10 µm brain section showing the total binding of the radiotracer, co-incubation with 10 µM GW405833 or reference compound (target region, *h*CB2R D80N, circled); and **e** homologues displacement study (autoradiography) for the estimation of the binding affinity of the radiotracer (n = 3)

The specific binding to the *h*CB2R, the ability to cross the blood–brain barrier and signal-to-background ratio (SUVr) of [^18^F]JHU94620-*d*_8_ in vivo was investigated in a rat model with a local overexpression of *h*CB2R(D80N) in the right striatum (target region) by dynamic PET studies (Figs. 4 and S11, Tables S1 and S2).

A constant radioactivity concentration with an SUV_mean_ of 7.5 ± 1.5 was observed in the *h*CB2R(D80N)-overexpressing target region starting at 10 min after radiotracer administration. In the potential reference regions (contralateral and cerebellum), TAC peaks with an SUV_mean_ of about 3 were reached at 3 min p.i., followed by a washout phase reaching an SUV_mean_ of 0.8 ± 0.3 in the contralateral and 0.16 ± 0.03 in the cerebellar region at 60 min p.i.. As a result of the radiotracer clearance from CB2R negative and low expressing brain regions, the increasing signal-to-background ratio over time reached an SUVr of 10 ± 4 utilizing the contralateral and 47 ± 4 utilizing the cerebellum as reference regions at the end of the observation period (Fig. 4a, b).

The reversible binding of [^18^F]JHU94620-*d*_8_ towards the human CB2R(D80N) receptor could be demonstrated in displacement studies (Fig. 4c, Table S2). The contralateral side is less suitable as a reference region, as the high spillover signal from the ipsilateral side led to an apparently strong underestimated SUVr (*h*CB2R(D80N)-to-contralateral) and overestimated blocking effect of the displacement compound (AUC_20-60min_ =—6.5%, Table S2). However, the signal in the cerebellum was not affected by the treatment over the observation time with GW405833 (*p*-values > 0.05, multiple unpaired t-test; Figure S11, Tables1 S2 and S2), underlining the eligibility for a reference region. Compared to the control group (Table S1), the signal in the target region was reduced 10 min after the injection of GW405833 (*p*-values < 0.05, multiple unpaired t-test), reflected by the 61% reduced AUC_20-60min_ when normalized to the cerebellum (Table S2).

Interestingly, the subsequent homologous in vitro binding study (Fig. 4d, e) with cryosections of these brains revealed a *K*_D_ of 41 nM, indicating an 85 to 100-fold lower binding affinity of [^18^F]JHU94620-*d*_8_ for the *h*CB2R(D80N) compared to CB2R in rat spleen and CHO(*h*CB2R) cell membrane binding assay (Fig. 1c).

### Pharmacokinetic modelling

**Fig. 5:**
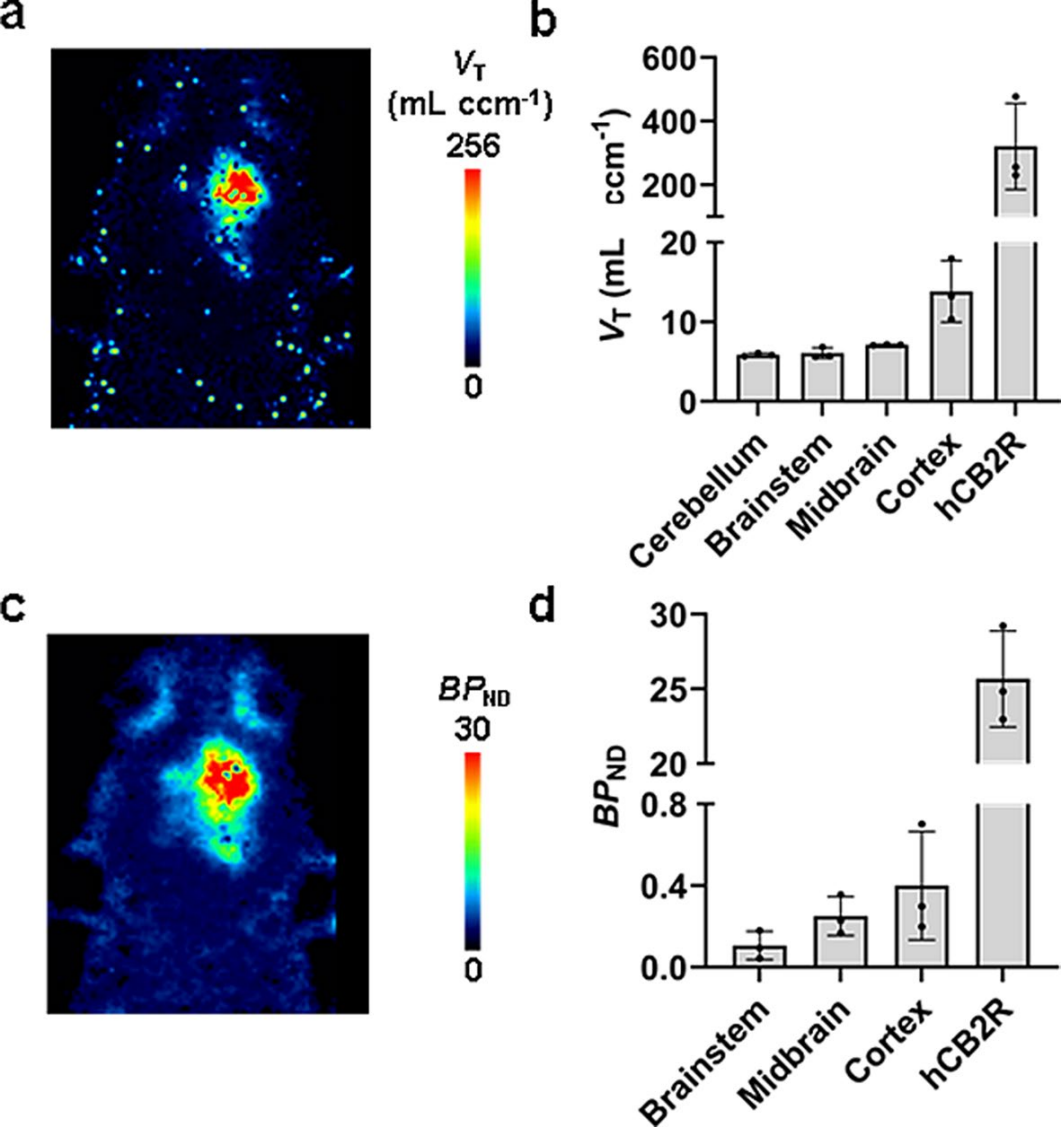
Estimation of the total volume distribution *V*_T_ and the binding potential *BP*_ND_ of [^18^F]JHU94620-*d*_8_ in the brain of rats with a local overexpression of the *h*CB2R(D80N) in the right hemisphere. **a** exemplary parametric *V*_T_ map in mL ccm^−1^ (two-tissue compartment modelling) of a coronal head plane; **b** comparison of the *V*_T_ in different brain regions (n = 3);**c**exemplary parametric *BP*_ND_ map (SRTM); and **d** comparison of the *BP*_ND_ in different brain regions (n = 3)

To quantify the uptake of [^18^F]JHU94620-*d*_8_ in the brain with the local overexpression of the *h*CB2R(D80N) the total volume distribution (*V*_T_) and binding potential (*BP*_ND_) were estimated (Fig. 5). From the rats used for in vivo metabolism studies a hematocrit of 0.56 ± 0.04, as well as a stable free plasma fraction (*f*_p_) between 5 and 60 min after administration of the radiotracer of 0.3 ± 0.1 (n = 11) was determined (*p*-value > 0.05, Figure S11). A population-averaged image-derived input function (pIDIF), determined in biodistribution studies was corrected for *f*_p_ and plasma-to-whole blood ratio and used for further pharmacokinetic modelling. The *V*_T_ in the brain region (Fig. 5a, b) bearing the *h*CB2R(D80N) protein overexpression was 320 ± 135 mL ccm^−1^and compared to the other investigated brain regions with a *V*_T_ value between 5.9 and 13.8 mL ccm^−1^ up to 23 times higher. Consequently, the *BP*_ND_ of 26, estimated by a SRTM (Fig. 5c, d) approach utilizing the cerebellar region as a reference, reflects the markedly elevated receptor expression in this rat model. Conversely, the *BP*_ND_ in the other investigated brain regions was observed to range between 0.1 and 0.4, indicative of the very low expression of the CB2R in the healthy brain.

## Discussion

In this study, we demonstrated an increased metabolic stability for [^18^F]JHU94620-*d*_8_ compared to its non-deuterated isotopologue [^18^F]JHU94620. The high binding affinity of [^18^F]JHU94620-*d*_8_ to the CB2R in vitro and in vivo with favourable pharmacokinetics was reflected by a CB2R-specific binding in the spleen of rats and high uptake in a brain region containing high CB2R(D80N) protein expression.

Radiometabolites that cross the BBB may complicate the quantification of target structures in the brain due to unpredictable signal contribution (Pike 2009; Ghosh et al. 2020). In the case of [^18^F]JHU94620, strategies, such as changing the position of the radiolabel or introducing potentially more stable moieties, negatively affected the binding characteristics to the CB2R (Gündel et al. 2022; Aly et al. 2021). Therefore, to increase the metabolic stability and preserve the binding properties of [^18^F]JHU94620, hydrogen atoms at its fluorine-18 labelled butyl chain were replaced by deuterium, as shown for other radiotracers (Lai et al. 2021; Haider et al. 2020). In this study, the formation of more hydrophilic BBB-crossing radiometabolites, presumably hydroxylated radiometabolites, was most markedly reduced for the isotopologue [^18^F]JHU94620-*d*_8_, as demonstrated in vivo*.* However, these radiometabolites could not be generated by the liver microsome in vitro assay and the metabolic pathways involved still need to be identified in detail, e.g. with regard to possible conjugation reactions (Phase II metabolism) (Testa and Krämer 2007). Moreover, since excretion is not covered in the liver microsome assays, the fraction of radiometabolites tends to be overestimated compared to in vivo. Although the metabolite pattern of [^18^F]JHU94620-*d*_8_ observed in liver microsomes assay in vitro was comparable for rat and human, the degradation by HLM was faster. Similar species differences were previously observed by us for benzothiazole-based JHU94620 derivatives in the liver microsome assay in vitro (Aly et al. 2021). Given that NADPH served as the sole cofactor in this study (Phase I radiometabolites), and thus conjugation reactions (Phase II radiometabolites) were not examined, the Phase I in vitro radiometabolite profile was comparable to that observed in vivo in rats. The structural analogue [^18^F]FC0324 published recently (Caillé et al. 2017; Auvity et al. 2024) showed comparable results regarding the biological evaluation and species-dependent metabolic stability in liver microsomal assays. However, in addition to its high CB2R binding affinity, it also exhibits a relevant CB1R binding affinity (*K*_i(CB1R)_ = 30 nM, *K*_i(CB2R)_ = 0.1 nM), which could potentially bias a pathological increased CB2R signal in regions in the brain with high CB1R density.

Autoradiography results demonstrated a CB2R-specific binding of [^18^F]JHU94620-*d*_8_ in CB2R-rich regions of the spleen (white pulps) of different species, as it was shown for [^3^H]CP55,940 and [^3^H]/[^18^F]-RoSMA-18 (Haider et al. 2020; Massi et al. 1997). However, CB2R density and binding properties of CB2R radiotracers are species-dependent. Govaerts et al. reported a *B*_max_ of 0.71 ± 0.02 in rat and 0.31 ± 0.03 pmol mg^−1^ protein for mouse spleen by using a [^3^H]CP55940 binding assay (Govaerts et al. 2004), which was about five to ten times higher compared to our study using [^18^F]JHU94620-*d*_8_. A species-dependent binding affinity was reported for different CB2R ligands (Soethoudt et al. 2017; Teodoro et al. 2023). However, the species-dependent binding affinity for [^18^F]JHU94620-*d*_8_ is comparable between the investigated species. The high lipophilicity of the radiotracer, which is assumed to be comparable to that of its non-deuterated isotopologue, with a LogD_7.4_ of 3.2 (Moldovan et al. 2016) probably explains the high signal-to-background ratio observed in spleen cryosections from different species (CB2R-specific binding between 25 and 48% of total binding). Compared to other ^18^F-labelled CB2R radiotracers, which lack CB2R-specific binding in murine spleen (Gündel et al. 2022; Teodoro et al. 2023, 2021; Ueberham et al. 2023), the binding specificity of [^18^F]JHU94620-*d*_8_ of 48% is comparable to that of [^18^F]RoSMA-18 (71%) in rat spleen(Haider et al. 2020). Our experiments showed that neither a CB2R-specific binding of [^18^F]JHU94620-*d*_8_ nor an increase in non-specific binding of HLM-derived radiometabolites was detectable in rat spleen cryosections. However, the two more hydrophilic BBB-crossing radiometabolites which might be a result of radiodefluorination were not formed in our liver microsomal assays, which is a limitation of this in vitro approach. In a previous study by Gado et al., PAM was demonstrated to enhance the binding affinity of [^3^H]CP55,940 to CB1 and CB2R (Gado et al. 2019). Conversely, in our investigation, a relatively weak competitive behaviour was observed for [^18^F]JHU94620-d8, indicating that the binding sites for both compounds are in close proximity or potentially identical.

The CB2R-specific binding of [^18^F]JHU94620-*d*_8_ in vitro was confirmed in biodistribution studies with rats showing a CB2R-specific uptake in the spleen. The spleen SUV_mean_ of about 0.5 and the SUVr(spleen-to-blood) of about 1.5 at late time points in the baseline and pre-blocking studies may be partially accounted for by non-specific binding of the radiotracer and spill-over signals from adjacent tissues, such as the intestine and stomach, as a consequence of a rather rapid hepato-biliary excretion.

Although a low *f*_P_ of [^18^F]JHU94620-*d*_8_ was determined, which is common for lipophilic radiotracers (Pike 2009), a sufficiently high uptake of [^18^F]JHU94620-*d*_8_ in the brain was observed. In particular, the brain TACs in the rat model containing a high expression of the *h*CB2R(D80N) demonstrated a CB2R-specific and reversible binding of [^18^F]JHU94620-*d*_8_ in the brain. The G-protein uncoupled AAV2/7-CaMKII0.4-intron-*h*CB2R(D80N) construct was designed as a reporter gene system for in vivo applications with a high expression of the targetable *h*CB2R protein (Vandeputte et al. 2011). Hence, the estimated high *V*_T_ and *BP*_ND_ values in the *h*CB2R(D80N) expressing brain region confirmed these high expressions, as well as the very low CB2R expression in the other evaluated brain regions (Latek et al. 2011; Du et al. 2023; Govaerts et al. 2004). It is noteworthy that a reduced binding affinity towards the *h*CB2R(D80N) in comparison to the overexpressed *h*CB2R of CHO cells homogenates, as well as rodent spleen, was observed. This finding gives rise to the question of whether [^18^F]JHU94620-*d*_8_ is expected to function primarily as an agonist. As observed for other GPCR agonists, it is possible that [^18^F]JHU94620-*d*_8_ may exhibit a high binding affinity for receptors coupled to G-proteins and a low affinity for uncoupled receptors (Colom et al. 2019), as here for the uncoupled CB2R(D80N). Also, specific binding to the allosteric binding site of the CB2R, as suggested by the competition studies with CB2R-PAM needs further investigations. However, to the best of our knowledge up to date an increased binding to pathological increased CB2R of an ^18^F-labelled CB2R-radiotracer was demonstrated only for [^18^F]RoSMA-18-*d*_6_ in a mouse model for cerebral ischemia (Ni et al. 2021) and postmortem on human amyotrophic lateral sclerosis (ALS) spinal cord tissues (Haider et al. 2023). Hence, the sensitivity of [^18^F]JHU94620-*d*_8_ to detect a pathologically increased CB2R density needs to be investigated in upcoming studies.

## Conclusion

[^18^F]JHU94620-*d*_8_ is a novel BBB-penetrant PET radiotracer with excellent CB2R binding properties and improved metabolic stability. The rapid washout from the brain allows a potentially high target-to-background ratio, a prerequisite for the quantification of CB2R expression. The radiotracer can be prepared with high A_m_ and good radiochemical yields. Therefore, further characterization of [^18^F]JHU94620-*d*_8_ in CB2R-overexpressing pathological animal models and human tissue samples is highly recommended as a next step towards clinical translation.

## Supplementary Information

Additional file 1.

## Data Availability

The datasets generated and/or analyzed during the current study are available from the corresponding authors on reasonable request.

## Abbreviations

^1^H-NMR: Proton nuclear magnetic resonance
2-AG: 2-Arachydonilglycerol
AEA: Anandamide
AUC: Area under the curve
BBB: Blood-brain barrier
BCA: Bicinchoninic Acid
*BP*_ND_: Binding potential
CB1R: Cannabinoid Receptor 1
CB2R: Cannabinoid Receptor 12
CBD: Cannabidiol
DMF: Dimethylformamide
EA: Ethyl acetate
ECS: Endocannabinoid system
ESI: Electrospray ionization
Et_3_N: Triethylamine
EtOH: Ethanol
*f*_p_: Free plasma fraction
GPCR: G-protein coupled receptor
hCB2R: Human CB2R
HLM: Human liver microsom
HPLC: High performance liquid chromatography
HRMS: High-resolution mass spectrometry
MeCN: Acetonitrile
NADPH: β-Nicotinamide adenine dinucleotide 2′-phosphate reduced tetrasodium salt
NH_4_OAc: Ammonium acetate
OSEM3D: Ordered Subset Expectation Maximization applied to 3D
p.i.: Post injection
PAM: Positive-allosteric modulator
PBS: Phosphate buffered saline
PE: Petroleum ether
PET: Positron emission tomography
pIDIF: Population averaged imaging derived input function
RCC: Radiochemical conversion
RCP: Radiochemical purity
RCY: Radiochemical yield
RLM: Rat liver microsome
RP-HPLC: Reversed phase-HPLC
SRTM: Simple reference tissue modelling
SUV: Standardized uptake value
SUVr: SUV ratio
TAC: Time activity curve
TBAF: Tetrabutylammoniumfluorid
THC: Δ^9^-*Trans*-Tetrahydrocannabinol
THF: Tetrahydrofuran
TLC: Thin layer chromatography
t_R_: Retention time
TsCl: Toluenesulfonyl chloride
*V*_T_: Total tissue distribution volume
