# Supplementary material for: Development and evaluation of deuterated [18F]JHU94620 isotopologues for the non-invasive assessment of the cannabinoid type 2 receptor in brain
Source: EJNMMI Radiopharm Chem. 2024 Dec 23;9:91. doi: 10.1186/s41181-024-00319-2 (PMC11666850; doi:10.1186/s41181-024-00319-2)
Supplement: Supplementary file 1 — Additional file 1. [file 41181_2024_319_MOESM1_ESM.docx]

**Supplemental Information (SI)**

**Development and Evaluation of Deuterated [^18^F]JHU94620 Isotopologues for the Non-invasive Assessment of the Cannabinoid Type 2 Receptor in Brain**

**Authors:**

Daniel Gündel ^a, *^, Mudasir Maqbool ^b^, Rodrigo Teodoro ^a, c^, Friedrich-Alexander Ludwig ^a^, Anne Heerklotz ^a^, Magali Toussaint ^a^, Winnie Deuther-Conrad ^a^, Guy Bormans ^d^, Peter Brust ^a, e^, Klaus Kopka ^a, f^, Rareş-Petru Moldovan ^a, *^

**Affiliations:**

^a^ Helmholtz-Zentrum Dresden-Rossendorf, Institute of Radiopharmaceutical Cancer Research Department of Neuroradiopharmaceuticals, 04318 Leipzig/01328 Dresden, Germany;

^b^ Molecular Imaging Branch, National Institute of Mental Health, National Institutes of Health, 10 Center Drive, Bldg. 10, Rm B1D43, Bethesda, MD, 20892-1026, USA

^c^ Life Molecular Imaging GmbH, 13353 Berlin, Germany

^d^ Radiopharmaceutical Research, Department of Pharmaceutical and Pharmacological Sciences, KU Leuven, BE-3000 Leuven, Belgium

^e^ The Lübeck Institute of Experimental Dermatology, University Medical Center Schleswig-Holstein, 23562 Lübeck, Germany

^f^ Faculty of Chemistry and Food Chemistry, School of Science, TU Dresden, 01069 Dresden, Germany


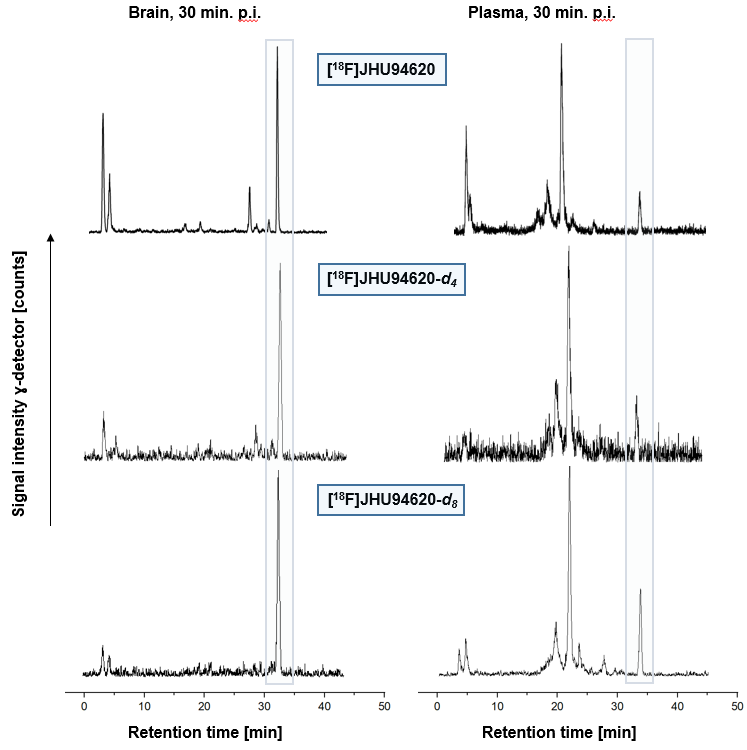


Figure S1: *In vivo* metabolism. Representative radio-HPLC chromatograms (method A) of tissue and organ samples of female CD-1 mice obtained at 30 min p.i. [extraction with MeOH/H_2_O (9:1); extraction yield: >94%].


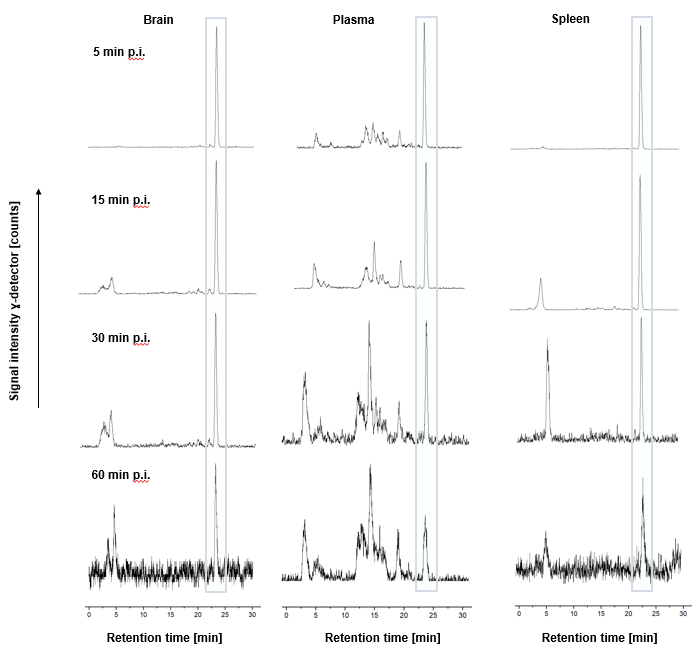


Figure S2: *In vivo* metabolism. Representative radio-HPLC chromatograms (method B) of tissue and organ samples of rats obtained at 5, 15, 30 and 60 min p.i. [extraction with MeOH/H_2_O (9:1); extraction yield: >94%].

**
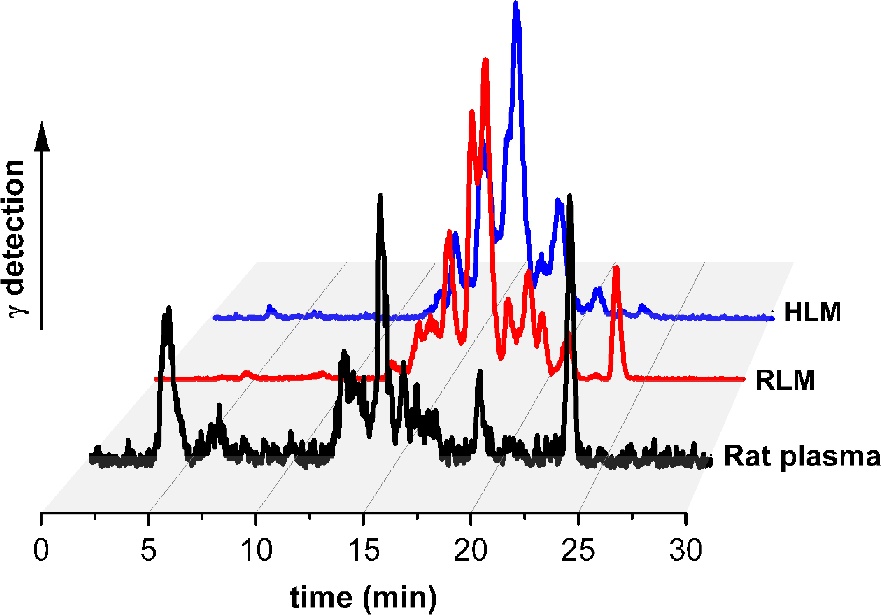
**

Figure S3: Comparison of *in vivo* and *in vitro* radiometabolite patterns of [^18^F]JHU94620-*d*_8_ (t_R_= 23.2 min). Representative radio-HPLC chromatograms (method B) of samples from rat plasma (60 min p.i.), as well as from rat (RLM) and human (HLM) liver microsomes after incubation in presence of NADPH, for 60 min each.

**
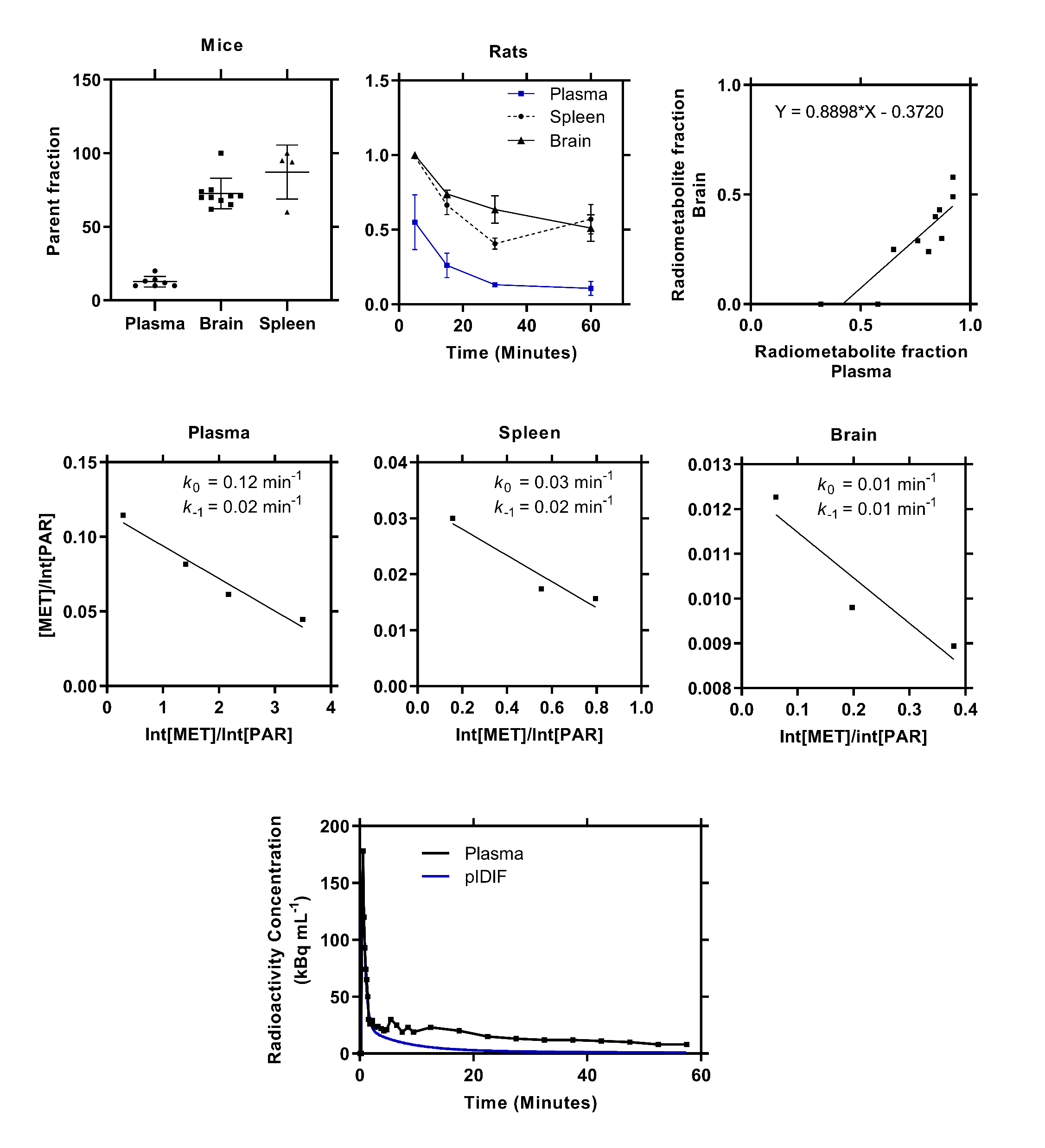
**

Figure S4: Plasma-brain radiometabolite plot. Linear regression analysis of radiometabolite distribution between blood plasma and brain (r² = 0.77).


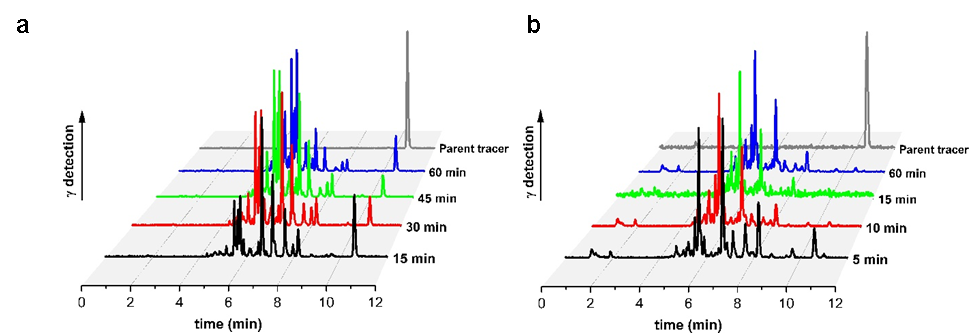


Figure S5: Comparison of the radiometabolite patterns detected for metabolic depletion of [^18^F]JHU94620-*d*_8_ *in vitro*. Representative radio-HPLC (method C) chromatograms of samples from RLM (a) and HLM (b) at different time points.

**
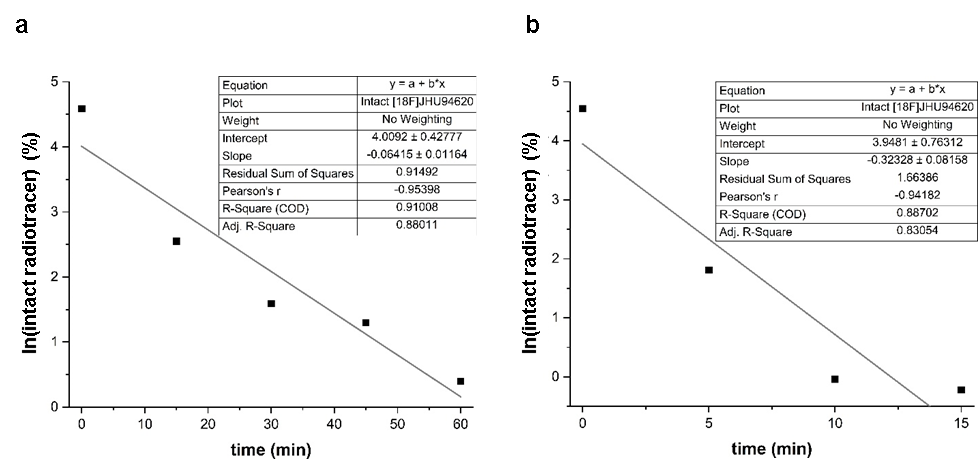
**

Figure S6: Graphical analysis of time-dependent depletion of [^18^F]JHU94620-*d*_8_ *in vitro* and linear regression for calculation of *in vitro* half-life (*t_1/2_*) for RLM (a) and HLM (b) using the following equations: *ln(intact radiotracer in %) = ln100 – slope × t,* and *t_1/2_ = ln2 / slope.*

**
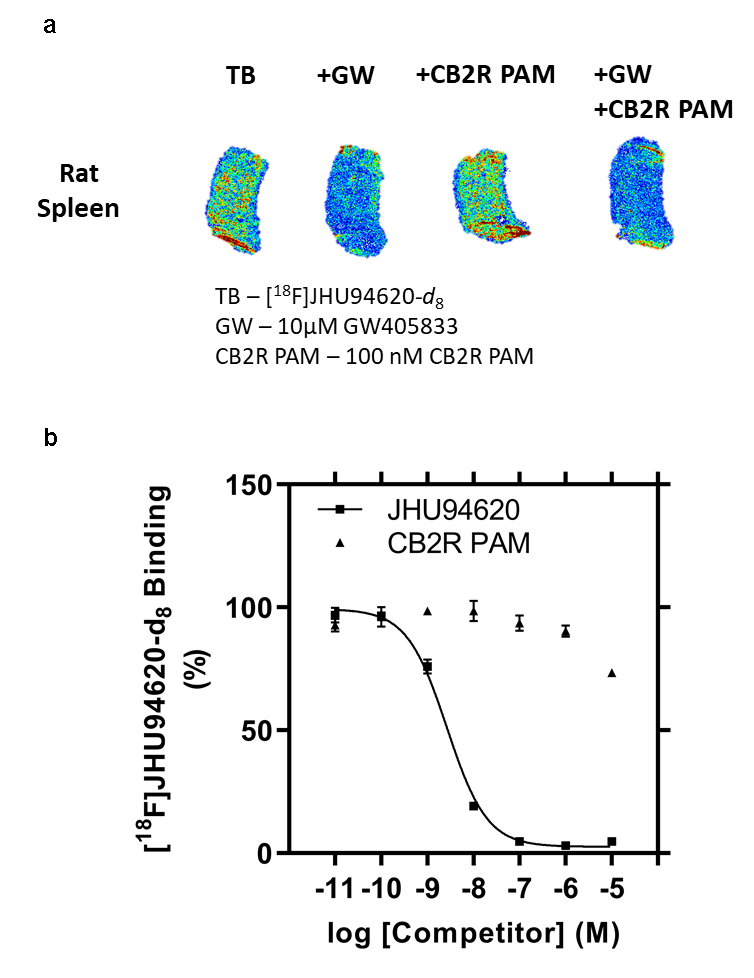
**

Figure S7: Effect of the CB2R positive allosteric modulator (CB2R PAM) on the *in vitro* binding of [^18^F]JHU94620-*d*_8_ to rat and human CB2R. (a) Representative autoradiographic images of rat spleen cryosections (10 µm) showing total radiotracer binding without co-incubation of competitor (TB), with 100 nM CB2R PAM and/or 10 µM GW405833 (GW) under the same conditions, (b) homologous competition (JHU94620) or CB2R PAM binding assay with cell membrane isolates of CHO(hCB2R) (n = 3).

**
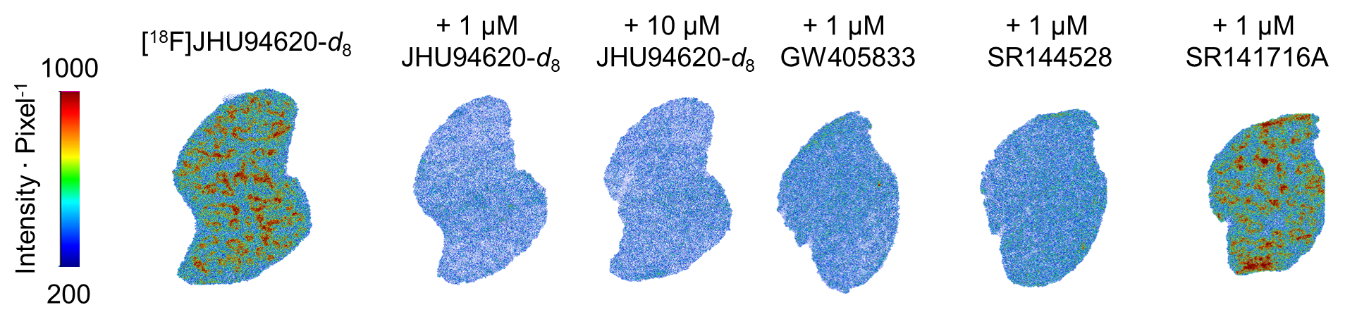
**

Figure S8: Exemplary autoradiography images of rat spleen cryosections (10µm) using [^18^F]JHU94620-*d*_8_ co-incubated with the reference compound (JHU94620-*d*_8_), the partial CB2R agonist GW405933, CB2R antagonist SR124528 or the CB1R antagonist SR141716A at given concentrations.

**
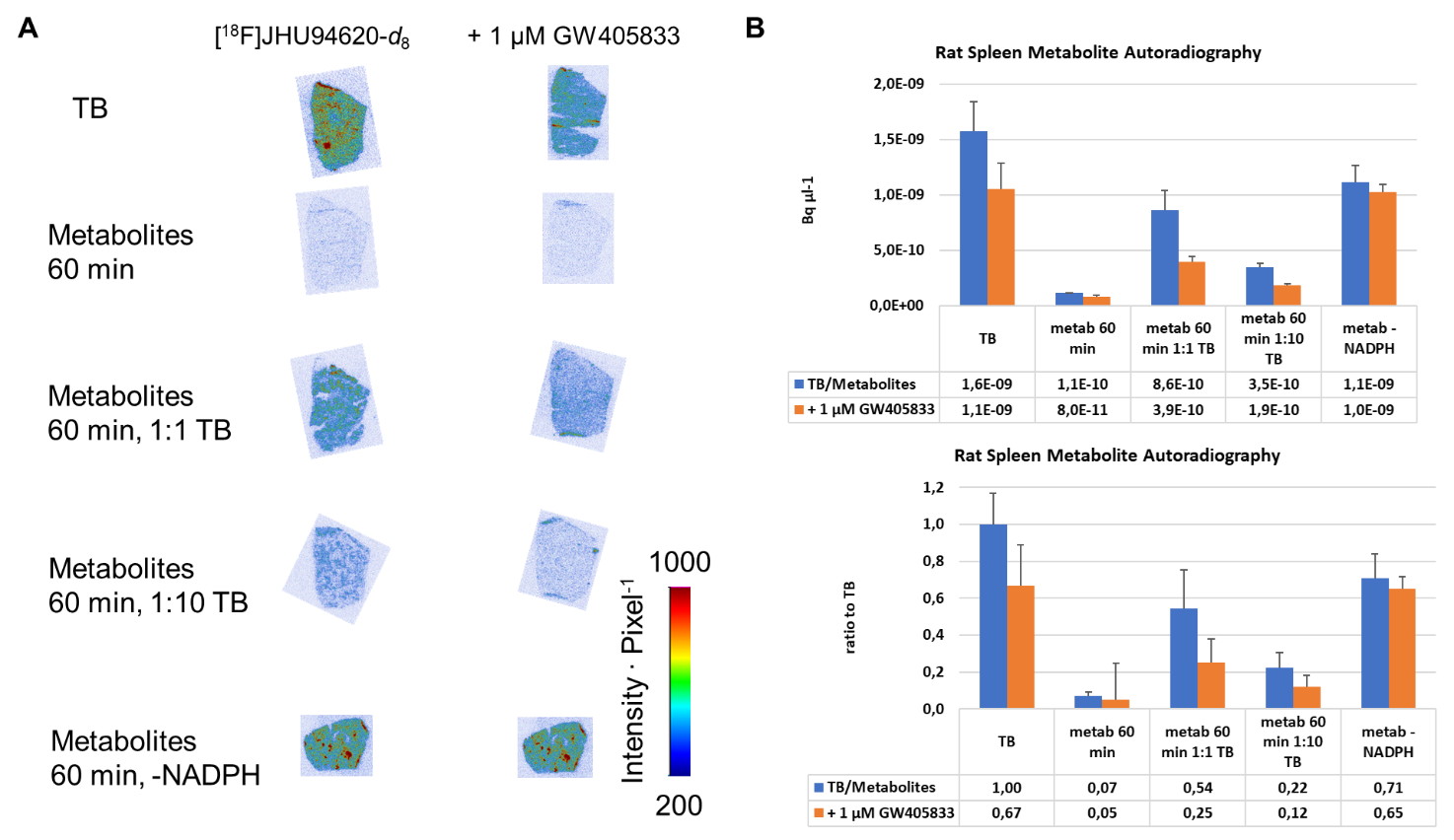
**

Figure S9: CB2R specific binding of [^18^F]JHU94620-*d*_8_ and radiometabolites of the radioligand derived after a 60 min incubation with human liver microsomes to rat spleen cryosections (10 µm). 200 kBq mL^-1^ was use of the radioligand alone (TB), only radiometabolites (Metabolites), a 1:1 or 1:10 mixture of TB and metabolites, as well as a metabolite assay negative control without using NADPH. (A) Exemplary autoradiography images and (B) spleen bound radioactivity concentrations in Bq mL^-1^ or bound ratio compared to TB (n = 3, mean ± SD).

**
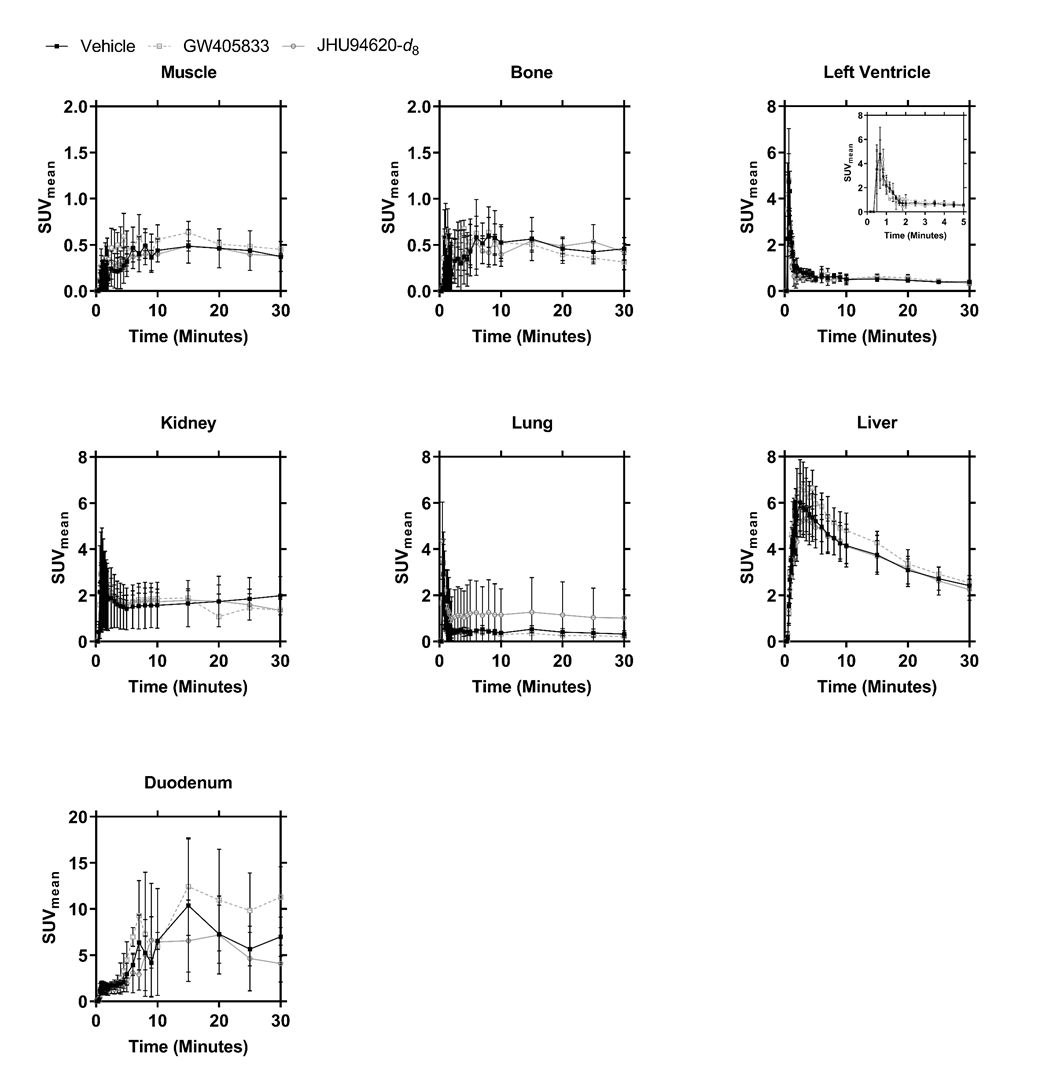
**

Figure S10: Biodistribution of [^18^F]JHU94620-*d*_8_ in Wistar rats derived by 30 min small-animal PET acquisitions under baseline (Vehicle) or with pre-blocking with 1.5 mg kg^-1^ bodyweight GW405833 or JHU94620-*d*_8_. Time–activity curves (TACs) for tissue uptake are expressed as mean standard uptake values (SUV_mean_), n = 3, mean ± SD.

Figure
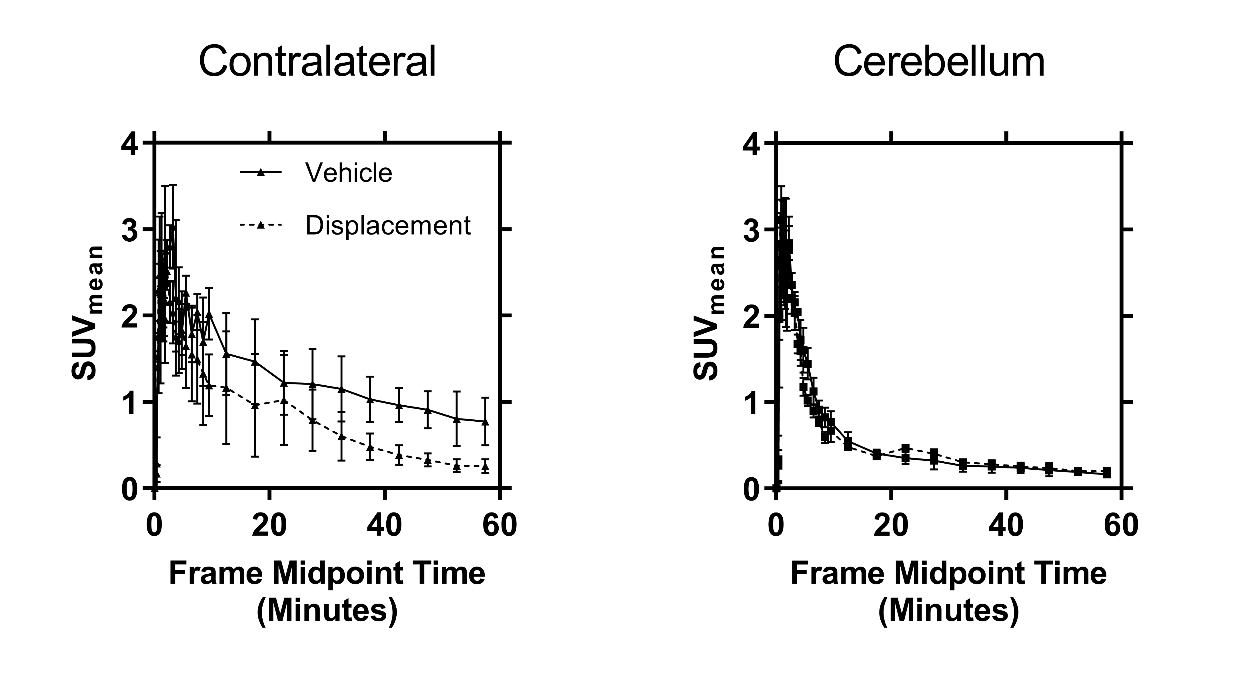
 S11: Time activity curves of the contralateral side and the cerebellum, with GW405833 (5 mg/kg, n = 3) and without (Vehicle) administered at 20 min post injection of [^18^F]JHU94620-*d*_8_ (n = 3; Mean ± SD).

Table S1. Non-compartmental (semiquantitative) analysis of TACs, obtained from the regions of local overexpression of the hCB2R(D80N) in the right striatum of rats, the contralateral region in the left striatum, and the cerebellum after i.v. injection of [^18^F]JHU94620-*d*_8_ (n = 3).

| TAC parameter | hCB2R(D80N) | Contralateral | Cerebellum | p-Value (Contralateral vs. Cerebellum) |
| --- | --- | --- | --- | --- |
| Time-to-peak  (min) | 10 | 3.5 | 2.5 | 0.054 |
| TAC peak value (SUV_mean_) | 7.5 ± 1.5  (10 to 60 min) | 3.3 ± 0.5 | 2.8 ± 0.2 | 0.148 |
| AUC  (SUV ∙ min) | 412 ± 90 | 74 ± 16 | 30 ± 4 | 0.218 |
| SUVr_60min_ hCB2R(D80N)-to- |  | 10 ± 4 | 47 ± 4 |  |

Mean ± SD; p-value - one-sided Student’s *t*-test; TAC – Time-activity curve, AUC -Area under the curve 0 to 60 min p.i.,

Table S3. Uptake of [^18^F]JHU94620-*d*_8_ into the hCB2R overexpressing right striatum normalized to the contralateral left striatum as well as cerebellum (SUV ratio, SUVr) with (n = 3) or without (vehicle, n = 3) administration of GW405833 (5mg/kg body weight, i.v.) at 20 minutes after radiotracer administration, expressed as area under the curve before (AUC_0-20min_) and after (AUC_20-60min_) injection of the interventional drug.

| hCB2R D80N-to- | Treatment | AUC_0-20min_ (CI_95%_)  in SUVr ∙ min | AUC_20-60min_ (CI_95%_)  in SUVr ∙ min |
| --- | --- | --- | --- |
| Contralateral | Vehicle | 67 (44 to 90) | 291 (226 to 355) |
|  | Displacement | 90 (60 to 121) | 272 (233 to 311)  - 6.5% |
| Cerebellum | Vehicle | 153 (136 to 170) | 1128 (1058 to 1198) |
|  | Displacement | 164 (150 to 178) | 435 (367 to 502)  - 61.4 % |

Mean; 95% confidence interval (CI_95%_).
